# Controlling Trapping, Release, and Exchange Dynamics of Micellar Core Components

**DOI:** 10.1021/acsnano.2c05144

**Published:** 2022-09-15

**Authors:** Rebecca Kaup, Aldrik H. Velders

**Affiliations:** † Laboratory of BioNanoTechnology, Wageningen University. Bornse Weilanden 9, 6708 WG Wageningen, The Netherlands; ‡ Interventional Molecular Imaging Laboratory, Department of Radiology, Leiden University Medical Center, 2300 RC Leiden, The Netherlands; § Instituto Regional de Investigacion Cientifica Aplicada (IRICA), Universidad de Castilla-La Mancha, Ciudad Real, 13071, Spain

**Keywords:** Exchange Dynamics, Complex Coacervate Core Micelles, Dendrimers, FRET, NMR, Self-Assembly, Responsive

## Abstract

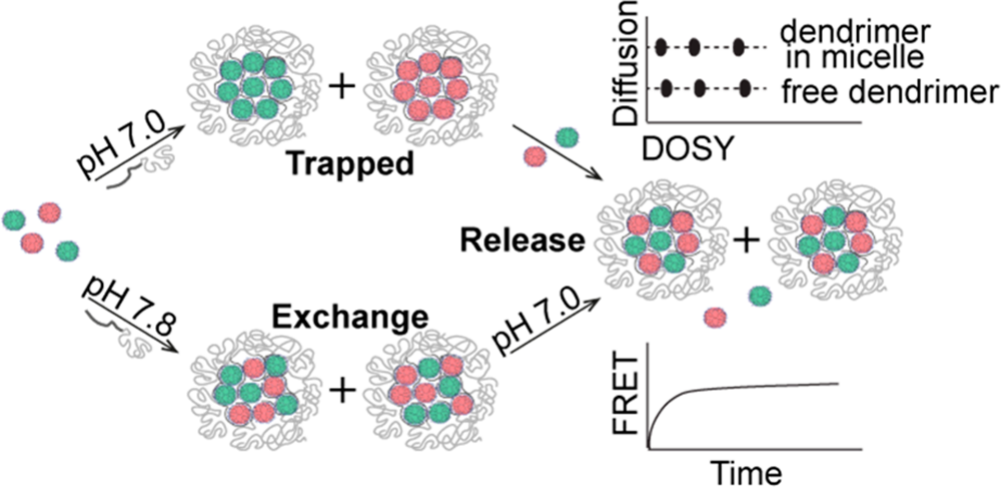

Whereas the formation and overall stability of hierarchically organized self-assembled supramolecular structures have been extensively investigated, the mechanistic aspects of subcomponent dynamics are often poorly understood or controlled. Here we show that the dynamics of polyamidoamine (PAMAM) dendrimer based micelles can be manipulated by changes in dendrimer generation, pH, and stoichiometry, as proven by NMR and FRET. For this, dendrimers were functionalized with either fluorescein (donor) or rhodamine (acceptor) and encapsulated into separate micelles. Upon mixing, exchange of dendrimers is revealed by an increase in FRET. While dendrimicelles based on dendrimer generations 4 and 5 show a clear increase in FRET in time, revealing the dynamic exchange of dendrimers between micellar cores, generation 6 based micelles appear to be kinetically trapped systems. Interestingly, generation 6 based dendrimicelles prepared at a pH of 7.8 rather than 7.0 do show exchange dynamics, which can be attributed to about 25% less charge of the dendrimer, corresponding to the charge of a virtual generation 5.5 dendrimer at neutral pH. Changing the pH of dendrimicelle solutions prepared at a pH of 7.8 to 7.0 shows the activated release of dendrimers. High-resolution NMR spectra of the micellar core are obtained from a 1.2 GHz spectrometer with sub-micromolar sensitivity, with DOSY discriminating released dendrimers from dendrimers still present in the micellar core. This study shows that dendrimer generation, charge density, and stoichiometry are important mechanistic factors for controlling the dynamics of complex coacervate core micelles. This knowledge can be used to tune micelles between kinetically trapped and dynamic systems, with tuning of exchange and/or release speeds, to be tailored for applications in, e.g., material science, sensors, or drug delivery.

Besides its ubiquitous importance in biological systems and materials science,^1^ self-assembly has proven to be an attractive and powerful strategy to create a variety of molecular superstructures, by design of building blocks allowing the tuning of physical–chemical parameters such as structure, size, shape, stability and responsiveness.^2−6^ However, the detailed characterization and understanding of the dynamic properties of such subcomponents within structures remains a great challenge, while this is important for the design, formation, and control of nanomaterials and their applications.^7,8^ Micelles coined complex coacervate core micelles or polyion complex micelles (C3Ms and PICs, respectively) are well-defined self-assembled systems formed by electrostatic interactions between a charged core building block and an oppositely charged–neutral block copolymer.^9−11^ Different core building blocks, from linear or branched polymers to proteins and DNA have been exploited.^10^ C3Ms are promising for nanomedical applications, including drug delivery, gene transcription, and contrast agents.^12^ For many applications the stability of micelles is an important factor and has been extensively investigated, with bulk solution characteristics such as critical micelle concentration (CMC) and critical salt concentration (CSC) being representative parameters.^13,14^ However, less research has been done on the dynamics, i.e. the exchange between core components of micelles, although both the exchange rate and the nature of exchange is crucial for e.g. cargo protection, as exchange of the material could lead to exposure of the cargo to the surrounding environment.^15^*In extremis*, one would like to have spatial and temporal control, from kinetically trapped systems, via controlled release, to dynamic exchange of subcomponents. A few recent studies and simulations on the exchange of C3M subcomponents show that the exchange rate is highly dependent on salt concentration and polymer length.^15−19^ However, it has not yet been shown that changing polymer lengths (charges per core component) can not only be used to control exchange rates but also can lead to kinetically trap micelles. Moreover, understanding such parameters would allow moving between kinetically trapped systems, controlled release, and exchange of subcomponents.

Dendrimers, and in particular polyamidoamine (PAMAM) dendrimers, are highly symmetrical, branched polymers with a defined size and number of surface groups.^20,21^ The number of surface groups doubles with increasing dendrimer generation, which in combination with their highly defined structure makes them very suitable for systematic studies. Furthermore, the voids of dendrimers can be used to encapsulate small molecules: e.g., drugs or nanoparticles.^22−24^ Dendrimers and dendrons are often used as building blocks for nanomaterials based on self-assembly and self-organization.^25,26^ In the past, we developed so-called dendrimicelles,^27^ which are formed by electrostatic interactions between positively charged PAMAM dendrimers and a neutral–negatively charged poly(methacrylic acid)-*b*-poly(ethylene oxide), pMAA_64_-*b*-pEO_885_, block copolymer.^28^ Encapsulation of dendrimer-encapsulated gold nanoparticles (AuDENs) was used to reveal the shape and aggregation number of dendrimicelles by cryo-transmission electron microscopy (cryoTEM).^28−31^ Recently, we proved the formation of multicompartment dendrimicelles with up to four different generation 6 dendrimers within one micellar core, by the combined use of cryoTEM and FRET.^32^

cryoTEM in particular has proven fundamental for investigating the stability of dendrimicelles in time, as the gold nanoparticles provide single-micelle information, revealing micelles based on generation 7 or higher PAMAM dendrimers to be kinetically trapped.^28^ We hypothesized that lower-generation dendrimers, i.e. with fewer charged surface groups, could form more dynamic micelle systems. Unfortunately, investigating the stability of dendrimicelles based on lower-generation dendrimers by cryoTEM is not straightforward, as PAMAM generations 5 and lower tend to form dendrimer-stabilized nanoparticle systems rather than dendrimer-encapsulated nanoparticles,^30^ inhibiting a systematic investigation of the exchange of individual dendrimers between the cores of different micelles. Moreover, TEM only allows visualization of snapshots at different time points, and the dynamics cannot be closely monitored over time. To allow investigation of this intriguingly well-defined and versatile class of dendrimicelles, we envisioned to exploit Förster resonance energy transfer (FRET) to study the exchange of dendrimers from the core of one dendrimicelle with dendrimers in the core of other micelles, as depicted in Scheme 1. Fluorescence and FRET are powerful techniques to study the thermodynamic properties and/or kinetics of self-assembled systems, including micelles.^33^ For FRET systems, solutions containing nanoassemblies with either a donor or an acceptor fluorophore are mixed, an increase in FRET efficiency revealing exchange of the building blocks or guest molecules.^16,17,34−36^ For the dendrimicelles, we show that the exchange is different for different dendrimer generations. Moreover, the exchange dynamics can be tuned by varying the generation of dendrimers used, by varying the pH, or by affecting the stoichiometry of the subcomponents.

**Scheme 1:**
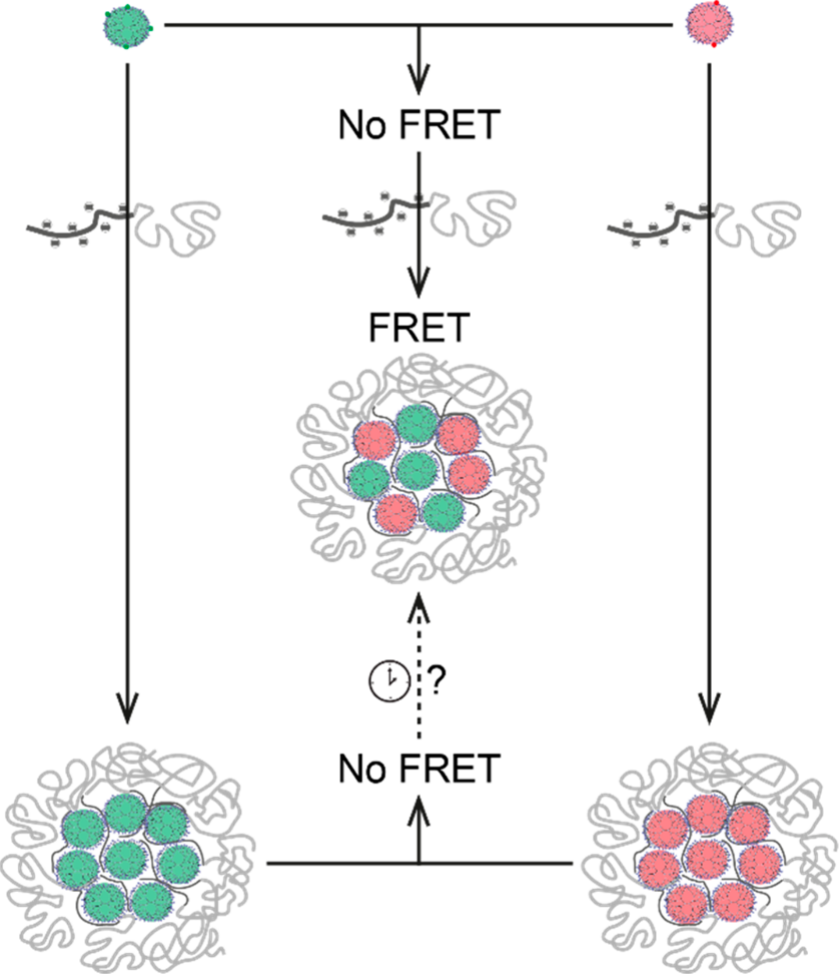
PAMAM Dendrimers Functionalized with either FITC (Green) or RITC (Red) and Consequently Used to Form Dendrimicelles by Mixing the Positively Charged Dendrimer Generation 4, 5, or 6 with a Neutral–Negatively Charged pMAA_64_-b-pEO_885_ Block Copolymer under Charge Stoichiometric Conditions To measure the exchange of dendrimers, micelles containing only the donor (FITC) or only the acceptor (RITC) are mixed at a 1:1 ratio. Exchange will result in micelles containing both donor and acceptor molecules, bringing them into close proximity and resulting in FRET. A premixed control sample containing the donor and acceptor gives the maximum FRET value corresponding to complete exchange. In this study different dendrimer generations and generation 6 at two different pH values or slightly off-stoichiometric conditions were tested and show control over exchange dynamics and trapping as well as activated release of dendrimers. For reasons of clarity, only generation 6 dendrimers are depicted in this scheme.

In a systematic approach, PAMAM dendrimers were functionalized with either fluorescein isothiocyanate (FITC) as a donor or with rhodamine B isothiocyanate (RITC) as an acceptor. As shown in Scheme 1, micelle solutions containing only the donor fluorophore or only the acceptor fluorophore were mixed, resulting in an increase in energy transfer upon exchange of dendrimers between micelles over time. FRET is an ideal methodology to prove exchange between micellar core components, as energy transfer is only possible when the donor and acceptor are in close proximity: i.e., within the same micelle core.^32^ In four different kinds of experiments we reveal the exchange properties of dendrimicelles based on dendrimer generations 4, 5, and 6, with, respectively, 64, 128, and 256 positive charges based on the theoretical maximum number of amine end groups. First, the formation of dendrimicelles is proven with DLS and nuclear magnetic resonance (NMR) spectroscopy. Consecutively, FRET reveals the exchange of donor- and acceptor-labeled dendrimers between the cores of micelles based on generations 4 and 5, but not for 6. Third, preparing dendrimicelles based on generation 6 at a higher pH, so that there is a lower overall charge per dendrimer, results in a G6-based dendrimicelle that rather than a kinetically trapped system that now is dynamic. In a final experiment, the surface charge of micellar core-dendrimers is altered by changing *in situ* the pH of the micelle solution, resulting in the release of dendrimers from the core. Whereas commonly micelles are investigated and characterized by light-scattering techniques, we here additionally introduce high-resolution solution NMR spectroscopy as an highly informative and powerful technique. Notwithstanding the enormous size and very low sub-micromolar concentrations of the micelles, aspects that normally preclude or strongly limit the use of NMR in micelle studies, the sensitivity of a 1.2 GHz spectrometer allows discriminative signals from dendrimers as micellar core subcomponents as well as free dendrimers. This work provides qualitative and quantitative tools to control the dynamics of subcomponents in complex hierarchically organized self-assembled structures, which is of use for the design of responsive materials with spatial and temporal control over the subcomponent dynamic properties.

## Results and Discussion

Here below, we first describe the formation and characterization of dendrimicelles based on three different generations of PAMAM dendrimers with a pMAA_64_-*b*-pEO_885_ block copolymer. Although the data are similar for dendrimicelles of generations 4, 5, and 6, the focus in this first part is on generation 6 alone for purposes of clarity. Importantly, we prove the invaluable use of high-field NMR spectroscopy, revealing the presence of dendrimers inside micellar cores. Second, based on a time-lapse FRET experiment we show that the use of generation 4 and 5 PAMAM dendrimers results in the formation of dendrimicelles that have dynamic core properties: i.e., the dendrimers can exchange between cores of different micelles. Differently, the G6-based system appears to be kinetically inert. Next, preparing dendrimicelles at a pH of 7.8 rather than 7 results in G6-based micelles that do show dynamic exchange of the dendrimers. Finally, the responsive release capabilities of the versatile dendrimicelle concept is proven by lowering the pH of a G6-based dendrimicelle prepared at high pH. Here again, NMR spectroscopy, and in particular diffusion-ordered NMR spectroscopy (DOSY), provides detailed and unambiguous insights.

### Formation and Characterization of Dendrimer-Based Micelles

**Figure 1:**
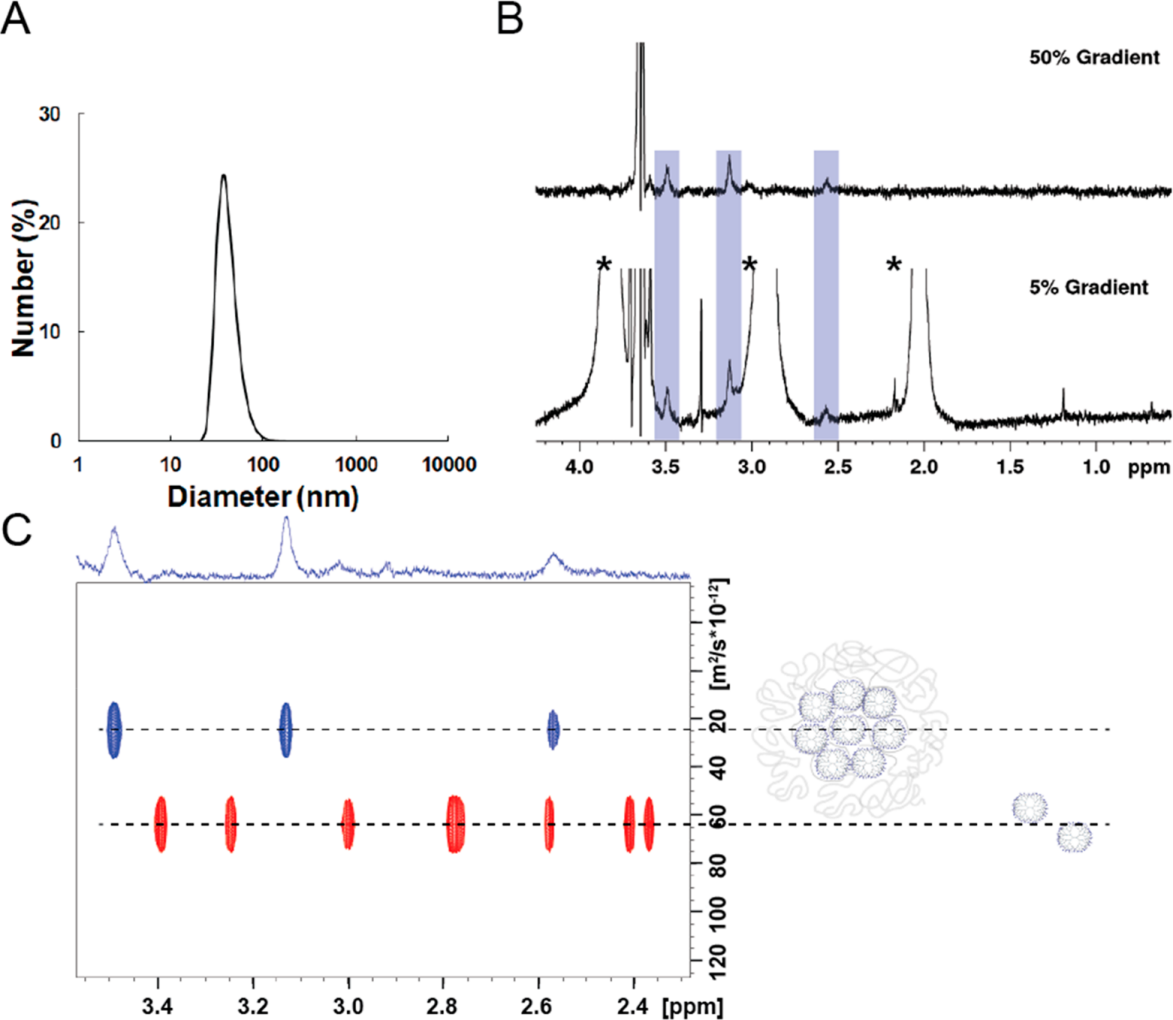
(A) DLS plot of dendrimer generation 6 based micelles. (B) ^1^H NMR 1D-DOSY spectrum of G6-based dendrimicelles with gradient strengths of 5% (lower spectrum) and 50% (upper spectrum). The peaks marked with an asterisk are from MOPS buffer. The peak at 3.6 ppm is from the PEG part of the block copolymer. The dendrimer peaks are highlighted by the blue bars in the two spectra, showing the dramatic improvement in spectral features of the dendrimers inside the micelle core when higher pulsed-field gradient strengths are applied. (C) Pseudo-2D DOSY overlay plots of generation 6 dendrimers encapsulated in micelles (blue) or free in solution (red), showing only the dendrimer signals. The 1D spectrum used on top of the 2D plot was taken with 30% gradient strength and shows the signals of the dendrimers inside the micelle core. The dashed lines are an aid to the eye, showing which peaks belong to the free dendrimers or to the dendrimers in the micelles (note that here the DOSY plot is an overlay of two separate pseudo-2D DOSY plots from two different samples).

First, different generations of PAMAM-based dendrimicelles were formed at pH 7 by mixing the dendrimers in a stoichiometric ratio with pMAA_64_-*b*-pEO_885_. Generations 4, 5, and 6 were chosen, because previous work already showed that generation 7 dendrimers form kinetically trapped dendrimicelles.^28^ We hypothesized that lower-generation dendrimers, i.e. with fewer charged surface groups, form more dynamic micelle systems. The micelles were characterized with dynamic light scattering (DLS), showing a hydrodynamic diameter of 40–60 nm, with generation 4 forming slightly larger micelles than generations 5 and 6 (Figure 1A and Figure S1). The critical micelle concentration (CMC) of the dendrimicelles was also determined with DLS. The CMC is <10 mg/L of the total polymer concentration for all three G4-, G5- and G6-based dendrimicelles (Figure S2).

Due to the micelle size (low tumbling times) as well as because of the very low (μM) concentrations, we had no high expectations of making the dendrimers residing inside the micelle core visible with NMR spectroscopy. However, with a state-of-the-art 28.2 T (1200 MHz 1H Larmor frequency) spectrometer, it appears possible to observe the dendrimers at sub-micromolar concentrations, even when they reside inside the micellar core (Figure 1B,C and Figures S3–S6). Clearly the mobility of the dendrimers inside the micellar core is high enough to allow sharp lines in NMR, and this is the case for micelles based on each of the three generations used. In particular, DOSY is a most useful technique and was used previously by us to investigate covalently functionalized dendrimers as well as a dendrimer-encapsulated-nanoparticle system.^37−39^ DOSY experiments are typically executed as a series of 1D echo experiments with an increasing pulsed-field gradient (PFG) strength.^40−43^ A single 1D DOSY spectrum acquired with a relatively high PFG strength acts as a kind of filter that selectively attenuates signals of small molecules more than the signals of larger components.^44^Figure 1B shows the spectra of generation 6 based micelles recorded with gradient strengths of 5% and 50%. At 50% gradient strength signals deriving from smaller molecules such as the buffer components present at high concentrations (20 mM of MOPS) are dramatically attenuated, while the peaks of larger particles (present at orders of magnitude lower concentrations, i.e. 2 μM of G6 PAMAM) remain visible. Therefore, at the rightly chosen gradient parameters, predominantly peaks of the block copolymer and the (micellar-core) dendrimers are observed. The fact that the dendrimers are detectable means that they can still move relatively freely or circulate inside the micelle core. In a cross-linked system such as dendroids,^45^ no dendrimer peaks could be detected. This shows that dendrimicelles are highly dynamic systems regarding the mutual movements and interactions of the subcomponents.

Figure 1C is a pseudo-2D DOSY plot showing the overlap of two different experiments, with the peaks of generation 6 dendrimers free in solution (red) compared to the dendrimers residing inside micelle cores (blue) (full DOSY plot of G6-based micelles is given in Figure S7). In comparison with free dendrimers, the peaks of encapsulated dendrimers shifted downfield, indicating the different environment inside micelle cores. In fact, the dendrimers are expected to be close in proximity to the negatively charged carboxylic acid groups of the block copolymer. Diffusion coefficients were obtained by fitting the attenuated signals of the series of 1D DOSY spectra, giving 5.25 × 10^–11^ and 1.02 × 10^–11^ m^2^/s for free dendrimers and dendrimers encapsulated in micelles, respectively. The higher diffusion coefficient for free dendrimers clearly corroborates that the other dendrimer peaks derive from dendrimers in a larger complex. The diffusion coefficients were further used to determine the micelle size. This was done by using the Stokes–Einstein equation (1), which shows an inversely proportional correlation between the diffusion coefficient and hydrodynamic radius
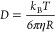
1with *k*_B_, *T*, η, and *R* being the Boltzmann constant, the absolute temperature, the viscosity, and the hydrodynamic radius, respectively. The self-diffusion and consequently the DOSY measurements are very susceptible to experimental parameters such as solvent, temperature, and hence viscosity, which can lead to relatively large errors in the determined diffusion coefficients in running absolute DOSY experiments or on comparing separate measurements.^37,46^ However, working under identical conditions or, even better, by measuring components in one and the same NMR tube, the exact calibration of the gradient coil, as well as temperature or viscosity control, is not of importance. For normalization of two components measured under the same conditions, all of the constants and parameters can be eliminated on equalizing the respective Stokes–Einstein equation, which results in eq 2, where the known diameter of the free dendrimer multiplied by the ratio of the diffusion coefficients of the dendrimers free in solution and inside the core yields the micelle dimension.
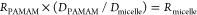
2

For our system, first the apparent hydrodynamic diameter of generation 6 PAMAM dendrimers (amine-terminated) was determined by using generation 6 PAMAM-OH dendrimers as an internal standard (Figure S8). The hydrodynamic diameter of the PAMAM-OH was used, as it is expected not to be affected by salt (double) layers; this is in contrast to the positively charged amine-terminated end groups of the regular PAMAM. Taking a size of 7.0 nm for generation 6 PAMAM-OH, the diffusion coefficient of the generation 6 PAMAM-NH_2_ dendrimer corresponds to a hydrodynamic diameter of 8.3 nm. When the diffusion coefficients of free generation 6 PAMAM dendrimers and generation 6 dendrimers inside a micelle core are consecutively compared, the ratio is 5.2. Using eq 2, this results in a micelle size of 43 nm in diameter, which corresponds well with the diameter found with DLS for generation 6 based micelles. So, in other words, the NMR signals of the G6 dendrimer, which apparently is highly mobile inside the micellar core, are clearly observable, and its diffusion coefficient then correlates to the hydrodynamic radius of the whole micelle in whose core it is residing. The micelle appears to be 5.2× larger in diameter than the dendrimer itself and corresponds with the dimensions obtained with DLS. In volume the micelle is then 140× larger than the PAMAM G6.

The diffusion coefficients of the free dendrimers of generations 4, 5, and 6 increase with decreasing dendrimer generation, as the dendrimer size is proportionally increasing with each generation. In contrast to this, the diffusion coefficient of the dendrimers is about the same for all three dendrimer generations in their respective dendrimicelle solutions, and moreover that small they indicate the dendrimers are residing inside the micelles (Figure S9). Also, the pseudo-2D DOSY plots corroborate that the micelle size is independent of dendrimer generation, in agreement with the DLS results and previous research.^27^ DOSY measurements have been used in micelle studies, e.g., to determine the critical micelle concentration (CMC) by detecting the core components free in solution while they were not visible once encapsulated in micelles^47^ or to study drug encapsulation in micelles.^48^ However, to the best of our knowledge, micellar core components of complex coacervate core micelles have not yet been detected and identified as residing inside the micellar core. For free dendrimers in solution typically eight signals can be discriminated, attributable to one set of four methylene groups from the repeating units of the dendrimer branches and one set of four methylene groups from the end ethyleneamide-ethylenamino group. Due to the high symmetry and branching nature of the PAMAM dendrimers, the intensities of these eight discernible methylene groups are close to identical,^37^ as can be seen also from the peaks in Figures S3 and S9. Interestingly, the methylene groups of the dendrimers residing inside the micellar core are not all equally well discernible. The peaks of interior methylene groups are more attenuated/broader than the terminal methylene groups (see Figure 1C, Figure S26, and the assignment of dendrimer peaks in Figure S22). This indicates a higher mobility of the terminal dendrimer parts while the interior parts are less mobile.

### Exchange of Dendrimers between Dendrimicelles

For the fluorescence measurements, amine-terminated PAMAM dendrimers of generations 4, 5, and 6 were functionalized as described previously,^32^ by reacting either FITC or RITC with dendrimers. A low functionalization degree, about 2–4 fluorophores per dendrimer, was chosen to keep a high charge density on the dendrimers to avoid interference with micelle formation and to minimize intramolecular quenching (see Table S1 and Figures S10 and S11). Dendrimicelles were then formed with each dendrimer generation at a ratio of 1:1 between FITC- and RITC-functionalized dendrimers. DLS showed sizes of 40–60 nm for all micelles (Figure S12), comparable to micelles formed with nonfunctionalized dendrimers (Figure 1A and Figure S1). Likewise, optical spectral properties are not significantly influenced by micelle formation and no intermolecular quenching was observed (Figures S13–15).

In order to measure the exchange of dendrimers, micelles containing either only FITC-functionalized dendrimers (donor) or only RITC-functionalized dendrimers (acceptor) were formed. Micelle solutions were left overnight to make sure that the systems were at equilibrium and subsequently mixed at a 1:1 ratio between donor and acceptor micelles. Thus, only the exchange was studied, not the micelle formation process. The emission of the donor and acceptor upon donor excitation at 480 nm was measured every 1 min for 2 h. The ratio between acceptor and donor emissions (575–615 nm:500–540 nm) was followed to study the exchange. An increase in the acceptor:donor (A/D) ratio indicates more energy transfer and therefore an increase in proximity of donors and acceptors, indicating that the exchange of dendrimers between micelles occurs. As shown above in the NMR discussion, dendrimers can still freely move despite encapsulation into micelle cores, meaning that fluorescence measurements represent an average distance between donor and acceptor fluorophores.

First, two separate samples were measured: i.e. dendrimicelles based on only donor-labeled dendrimers and dendrimicelles based on only acceptor-labeled micelles. The sum of the emission spectra of these two reference samples was taken as a starting point for the exchange experiments (0% FRET). Likewise, a premixed sample containing the donor and acceptor within the same micelle core in a 1:1 ratio was used as a reference for complete exchange (100% FRET). The fraction of maximal FRET, α_A/D_, was determined by normalizing the measured A/D ratio in the exchange sample to the three reference samples, using eq 3
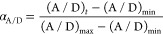
3with α_A/D_ being a dimensionless parameter^6^ and (A/D)_min_, (A/D)_max_, and (A/D)_*t*_ being the acceptor/donor ratio before mixing (sum of only donor and only acceptor sample), that of complete exchange (premixed sample), and that from the exchange sample at time *t*, respectively.

**Figure 2:**
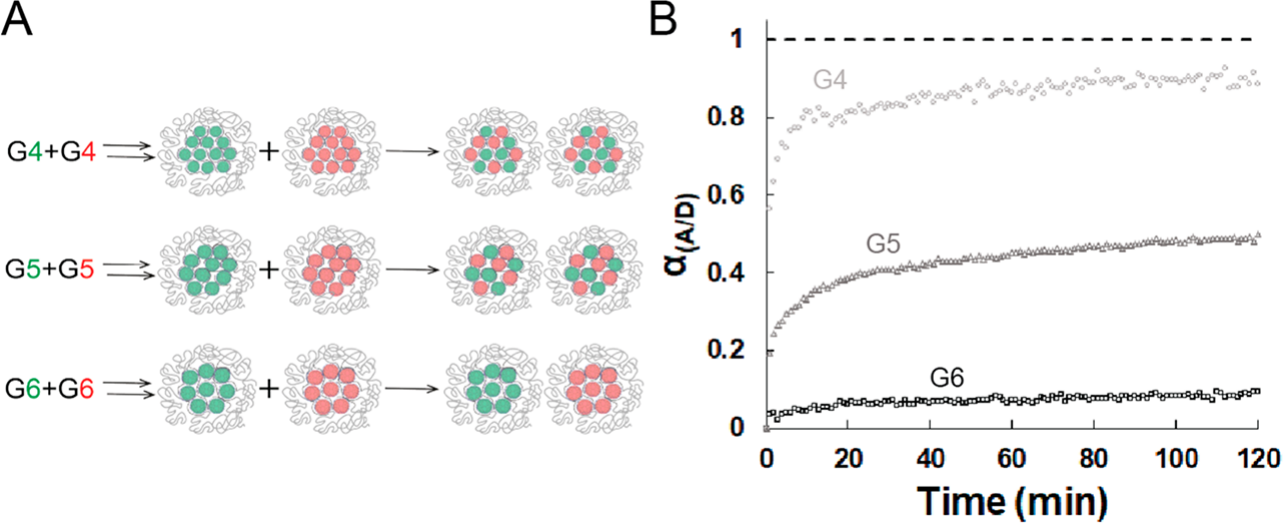
(A) Cartoon showing three different dendrimicelle samples. Generation 4, 5, and 6 dendrimers are encapsulated in either only donor (green) or only acceptor (red) micelles. The number of dendrimers per micelle increases with decreasing dendrimer generation. Different dendrimer generations are indicated by different sizes. Upon mixing, exchange of dendrimers between micelles leads to mixed micelles containing both donor and acceptor. For generation 6 almost no exchange could be observed and therefore the donor and acceptor stay in separate micelles. (B) α_A/D_ over time forgeneration 4 (light gray, circles), generation 5 (dark gray, triangles), and generation 6 (black, squares) based micelles. The first point of each line at α_A/D_ = 0 corresponds to the combined donor only and acceptor only spectrum measured for the respective dendrimer generation system. The dashed line at α_A/D_ = 1 corresponds to the spectrum of micelles containing both donor and acceptor, representing complete exchange; also here, for each dendrimer generation system the respective control samples were measured with the corresponding generation of dendrimer. Excitation was at 480 nm.

Micelles were formed containing generation 4, 5, or 6 dendrimers labeled with either donor or acceptor fluorophores and were consecutively mixed and monitored over time (Figure 2A). For generation 6 based micelles, α_A/D_ hardly changes within 2 h (Figure 2B), indicating a kinetically trapped system or at least very slow exchange. This is probably due to the high amount of charged surface groups in higher generations: i.e., theoretically 256 for generation 6. In contrast to that, in generation 4 based systems FRET increases rapidly (Figure 2B). A sharp increase takes place in the first 5 min, followed by a slow increase reaching the maximum value, and therefore close to complete exchange, after about 90 min. Generation 5 shows an exchange efficiency between generations 6 and 4. Again, a fast increase in the first 20 min can be seen. After that it is increasing slowly, reaching about 50% exchange after 2 h. A longer measurement shows that, also after 12 h, only about 50% of maximum exchange was reached (Figure S16). However, when a control sample was measured for 12 h, with premixed donor and acceptor present in the same micellar core, a bleaching effect could be detected after about 4 h (Figure S17). Therefore, it is important not to overinterpret the FRET data after the first 4 h in Figure S16, as they can be affected by the bleaching process.

For generation 5, in total three different micelle concentrations were tested: i.e., with 0.9, 0.45, and 0.23 μM of G5 concentration (Figure S18). As the exchange seems to be independent of concentration, the main exchange mechanism is probably expulsion and insertion, meaning that single polymers or small neutral clusters are expulsed from one micelle followed by insertion into another micelle.^16^ Still, this does not explain the two observed exchange rates: i.e., the initial fast increase in α_A/D_ and the following slower increase. So, whereas expulsion and insertion seems to be the main exchange process, other processes can also take place. Hollapa et al. got similar results for polyelectrolyte complexes; they identified the fast exchange as expulsion and insertion and the slower exchange as fusion and fission, which is the splitting and consequent merging of micelles.^49^ However, as we see a logarithmic increase over time (Figure S19), according to Lund et al. this cannot be attributed to only two exchange mechanisms but is better explained by a broad distribution of many different exchange rates.^50^ They showed that defects in the polymer structure as well as differences in activation energy and hierarchical constrained dynamics could play a role. Hierarchical constrained dynamics could imply for dendrimicelles that dendrimers positioned at the outer side of the micellar core exchange faster and more often than dendrimers in the center of the core structure, as more rearrangements are needed for that to happen. Therefore, the exchange process of dendrimicelles remains a very complex process but according to our data consisting of predominantly expulsion and insertion pathways, yet other processes might play minor roles as well.

A first-order reasoning to explain the difference between the exchange dynamics of micelles based on the three different generations is the number of charges per dendrimer. Generation 4 and the block copolymer pMAA_64_-*b*-pEO_885_ have the same number of charges at the tested pH 7.0 (theoretically 64). So, in theory, only one block copolymer can be attached to one G4 dendrimer, making it relatively easy for the dendrimer–block copolymer system to leave a micelle as a small neutral complex. For C3Ms in fact this is more favorable than exchange of single polyelectrolyte unimers.^19^ Generation 6, in contrast, has 256 charges, meaning that in theory at least four block copolymers should bind to one dendrimer to compensate for all the positive charges. As this would probably require more rearrangements of electrostatic bonds, the expulsion rate is decreased, making it more difficult to leave the micelle.^19^ Generation 5 has 128 charges and is an intermediate between G6 and G4 and indeed behaves accordingly. This suggests that the surface charge and multivalent charge interactions are important for the exchange rate of dendrimers between micelles. However, this is not a complete description, as between generations of dendrimers not only the number of end groups is changing but also the charge density: i.e., the charges per dendrimer surface and volume.^51^ As a rough estimate, considering the diameter of the dendrimers, the dendrimer surface increases by a factor of 2 on going from G4 to G6 PAMAM, while the charge quadruples. Thus, the charge density becomes higher on moving to higher generations, which will have its effects on the efficiency of interactions of the block copolymer with the dendrimer. Such discussions need to be taken with caution, as for higher generations defects are also more commonly found, such as missing or back-folding of part of the branches,^52^ obviously affecting the charge density.

Next, a mixture of different dendrimer generations within one micelle core was prepared. Generations 4 + 5, 4 + 6, and 5 + 6 were studied (Figure S21A). For each experiment, both generations are present within the same micelle, meaning that e.g. FITC-functionalized G4 and G5 are present in the donor micelle and RITC-functionalized G4 and G5 are present in the acceptor micelle. Micelle formation was confirmed by DLS for all three combinations (Figure S20) and the exchange monitored (Figure S21B). The combination G4 + G5 shows a fast exchange, reaching about 80% exchange after 2 h. This is close to what was seen for only G4, although the initial rapid increase is slower for the mixture than for only G4-based dendrimicelles, finished after 15 min and after 5 min, respectively. The mixtures of generations 4 and 6 as well as of 5 and 6 show an intermediate between the exchange rates for the respective single generations seen in Figure 1B. Generations 4 and 5 are still exchanging between micelles, leading to an increased α_A/D_, but the exchange rate is slower compared to only generation 4 or 5 micelles. The data from a previous section and this section show that the exchange rate and hence the stability of dendrimicelles can be tuned by using different single-dendrimer generations or a mix thereof. At the same time, these data also prove that different dendrimer generations can be encapsulated within one micelle core, in line with our previous work in which differently functionalized dendrimers of the same generation were incorporated inside a single micelle core.^32^

### Exchange of G6 Dendrimers between Micelles Prepared at pH 7.8

**Figure 3:**
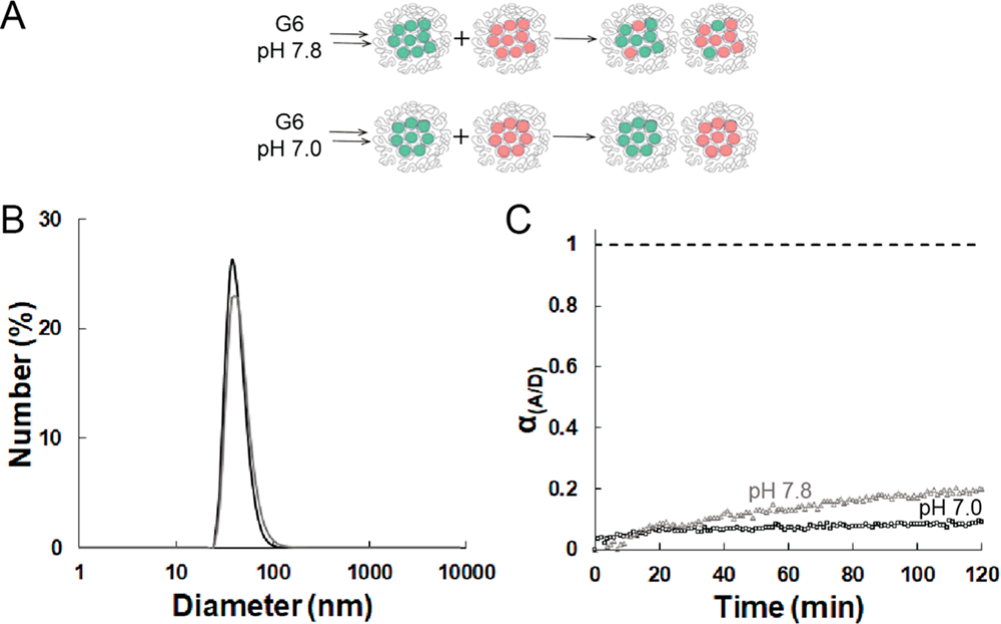
G6-based dendrimicelles formed at either pH 7.0 or pH 7.8 under stoichiometric conditions. (A) Cartoon showing the two different micelle samples. Micelles containing either a donor or acceptor were mixed in a 1:1 ratio. Exchange of dendrimers leads to mixed micelles containing donor as well as acceptor fluorophores. As previously observed, generation 6 at pH 7.0 does not exchange. (B) DLS plot of G6-based micelles at pH 7.0 (black) and 7.8 (dark gray) containing both donor and acceptor in a 1:1 ratio. (C) α_A/D_ over time for generation 6 at pH 7.8 (gray, triangles) and generation 6 at pH 7.0 (black, squares). The data points from generation 6 at pH 7.0 are repeated from Figure 2B for comparison. The first point of each line at α_A/D_ = 0 corresponds to the sum of a donor-only and an acceptor-only spectrum. The dashed line at α_A/D_ = 1 corresponds to the spectrum of micelles containing both a donor and acceptor, representing the maximum exchange. The excitation wavelength was 480 nm.

In the experiments described thus far, dendrimicelles were formed at pH 7.0 as the dendrimers and the carboxyl groups of the block copolymer are positively and negatively, respectively, charged at this pH value. At higher pH values the dendrimer surface charge decreases as fewer primary amines are protonated. As from the previous experiments it was clear that the charge and probably charge density play an important role in the exchange dynamics, we envisioned to use this to stimulate the exchange of higher-generation dendrimers. Dendrimicelles are stable in a pH range of ∼6 to ∼8;^28^ thus, we decided to measure the exchange of generation 6 dendrimers in dendrimicelles prepared at a pH of 7.8 (Figure 3A). Importantly, at this pH not all of the primary amines of a dendrimer are protonated and actually the overall charge is about 25% lower than that of a completely charged generation 6 dendrimer.^53^ A generation 6 dendrimer with only 75% of its maximum charges has about 192 charges, which corresponds to a virtual kind of generation 5.5 PAMAM at pH 7.0, considering only the surface charges. The increase in pH is not expected to influence the charge of the block copolymer, with the p*K*_a_ of the carboxylic acid groups being ∼4.5. In order to avoid effects related to changing the pH during or after mixing, the dendrimicelles were formed already at pH 7.8, at the theoretical charge stoichiometric conditions at that pH. Figure 3B shows that the size is similar to that of a G6-based dendrimicelle formed at pH 7.0, but more dendrimers are present per micelle, as we have less charge per dendrimer at this higher pH value.^54,55^Figure 3C shows the exchange dynamics of generation 6 at pH 7.0 and 7.8. The initial absence of FRET at pH 7.8 indicates a kinetically trapped G6 system, similar to what is observed for generation 6 dendrimicelles at pH 7.0. However, in contrast to the pH 7.0 data (see Figures 2B and 3C), at pH 7.8 an eventual clear increase in FRET is seen, about 20% after 2 h. Apparently, the G6 at pH 7.8, which correpsonds to a virtual generation 5.5 at pH 7, forms dendrimicelles at the borderline between kinetically trapped and (slow) exchanging. This is highlighted in the cartoon in Figure 3A, with the resulting dendrimicelles showing a mix of differntly labeled dendrimers in the core, but still a clear excess of either the donor- or acceptor-labeled dendrimers.

### pH-Responsive Release and Exchange of G6-Dendrimers from Dendrimicelles

**Figure 4:**
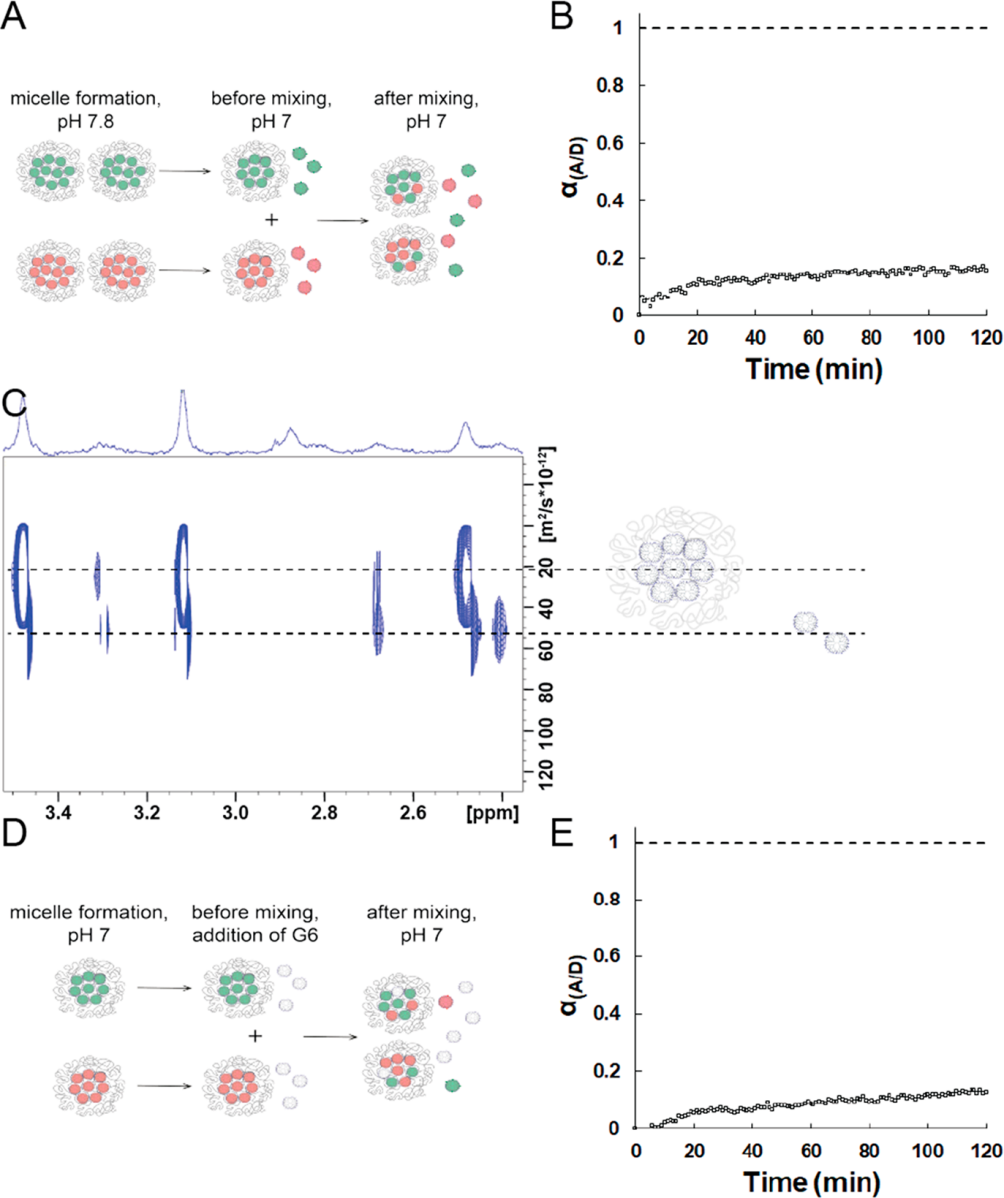
(A) G6-based micelles formed at pH 7.8 under stoichiometric conditions. Before mixing donor and acceptor micelles, the pH was decreased to 7.0, resulting in a nonstoichiometric condition leading to the realease of dendrimers. (B) α_A/D_ over time after changing the pH to 7.0. The first point at α_A/D_ = 0 corresponds to the sum of a donor-only and an acceptor-only spectrum. The dashed line at α_A/D_ = 1 corresponds to the spectrum of micelles containing both donor and acceptor, representing the maximum exchange. The excitation wavelength was 480 nm. (C) DOSY spectrum of an aged sample of nonfunctionalized G6-based dendrimicelles after changing the pH from 7.8 (stoichiometric) to 7.0. Only the dendrimer signals are shown. The 1D spectrum at the top part was taken with 30% gradient strength. Note: in contrast to the DOSY plot in Figure 1, here the free dendrimer and the dendrimer-inside-micelle are both present in the same NMR tube and this is a single DOSY experiment proving the release of dendrimers from micelle cores. (D) G6-based micelles formed at pH 7.0 under stoichiometric conditions. Before mixing donor–and acceptor micelles, 25% of unlabeled G6 dendrimers were additionally added, resulting in a nonstoichiometric condition. (E) α_A/D_ over time after addition of free dendrimers. The first point at α_A/D_ = 0 corresponds to the sum of a donor-only and an acceptor-only spectrum. The dashed line at α_A/D_ = 1 corresponds to the spectrum of micelles containing both donor and acceptor, representing the maximum exchange. The excitation wavelength was 480 nm.

Following up on the data obtained in the previous section, we hypothesized we could exploit the G6 dendrimer properties at different pH values to investigate the responsive release of dendrimers from a micelle core. After G6-based dendrimicelle formation at pH 7.8 under charge stoichiometric conditions, the pH was subsequently decreased to 7.0. This lowering of the pH results in an increase in the degree of protonation of the primary amine groups of the dendrimer and therefore results in an excess of positive charges in the micelle core with respect to the block copolymer charge. We hypothesized that this would lead to a rearrangement of the micelles aiming for charge stoichiometry. During this process dendrimers should then be released (Figure 4A, left part).

First, NMR measurements show that dendrimers are indeed released from the micelles after lowering the pH from 7.8 to 7.0 (Figure 4C). The DOSY plot clearly shows two types of dendrimers: i.e. dendrimers still encapsulated in micelle cores and released dendrimers free in solution. The released dendrimers show a higher diffusion coefficient and slightly upfield shifted signals. In theory, approximately 25% of the dendrimers could be pushed out of the micelles after decreasing the pH, corresponding to a free dendrimer concentration of about 0.6 μM. Additional proton and DOSY-filtered NMR spectra from the same sample before (Figures S23 and S24) and after (Figures S25 and S26) changing the pH to 7.0 confirm the release of dendrimers. At pH 7.0, extra peaks appear with a slight upfield shift, corresponding to released dendrimers, whereas these are not visible at pH 7.8. The peaks of the released dendrimers overlay with the peaks from dendrimers free in solution. Mostly the peaks labeled as C, c and D (highlighted by the black box in Figures S23–S26) are illustrative, as the other dendrimer peaks are influenced by overlapping residual MOPS buffer peaks. By comparing the peak height of dendrimers inside micelles and released dendrimers, a release of about 10–15% is estimated (peak at 3.47 ppm to 3.44 ppm, respectively). Furthermore, DLS measurements of the same micelle sample show a discrete decrease in scattering intensity after changing the pH from 7.8 to 7.0, additionally corroborating the release of dendrimers from micelles (Table S2). The observation of free G6 in solution proves that dendrimers are pushed out of the micellar core when the pH is changed from 7.8 to 7.0. This lowering of the pH causes an excess of positive charges inside the core, and the charge stoichiometry is then restored by the release of part of the dendrimers. By releasing part of the dendrimers into solution, the aggregation number of the dendrimicelles is then back to the “normal” one at pH of 7.0: i.e., 30 dendrimers per micelle for G6.^29^

Interestingly, complementary to the NMR data shown above that were obtained from a several days old sample, i.e. when dendrimers have been released from the micelles, the FRET experiment in Figure 4B shows an increase in time of the FRET in the first hours after lowering the pH. The FRET data indicate that there is exchange of the G6 dendrimers between micelle cores taking place now also at pH 7.0, whereas freshly prepared G6 dendrimicelles do not show such an exchange (Figure 2B). The exchange after 2 h is about 15–20% of the maximum FRET. Combining the conclusions from the NMR and FRET experiment, we can state that release of dendrimers is taking place; moreover, the excess of about 25% of dendrimers in solution with respect to the charge stoichiometry at pH 7.0 also results in the G6 dendrimers exchanging between micellar cores. To corroborate this, exchange between generation 6 based dendrimers was measured at pH 7.0 after adding 25% of (unlabeled) generation 6 dendrimers (Figure 4D). Again a change corresponding to an exchange of about 15–20% was visible after 2 h (Figure 4E), confirming that the addition of dendrimers can influence the kinetic stability of the cores of dendrimicelles and initiate slow exchange of dendrimers between generation 6 based dendrimicelles. As unlabeled dendrimer is added, the overall exchange is slightly higher than the corresponding 15–20% shown in the exchange described in the earlier experiments. On the one hand, from earlier FRET experiments it is known that the FRET efficiency remains unaltered when mixing in up to 30% of nonlabeled dendrimers into the core containing a mix of donor- and acceptor-labeled dendrimers.^32^ On the other hand, the release of also fluorophore-labeled dendrimers from micelle cores results in an overall lower α_A/D._

## Conclusion

We have presented the formation of dendrimicelles containing generation 4, 5, and 6 PAMAM dendrimers. NMR and especially DOSY measurements unambiguously prove that the dendrimers are residing inside the micelles for all tested generations and are still moving and/or rotating fast enough on the NMR time scale to allow high-resolution spectra. Dendrimers functionalized with either FITC or RITC were used to study the exchange of dendrimers between the cores of micelles by monitoring the change in FRET signal. The results showed that generation 6 based dendrimicelles are kinetically trapped, whereas generation 5 and 4 based micelles do show exchange of the dendrimers between micelles. The exchange rate of smaller generations is faster than that for larger dendrimer generations, meaning that probably the number of charges plays a major role in determining the speed of exchange events. Thus, the exchange rate can be tuned from kinetically trapped systems to fast dynamics using different dendrimer generations, and further also by mixing two different generations within the same micelle core. So far only mixtures of two different generations and a mixing ratio of 1:1 between generations based on the number of dendrimers have been studied. Different ratios could even lead to more control of exchange rates. As the exchange rate is independent of concentration, expulsion and insertion are the dominant exchange mechanisms for dendrimicelles. Furthermore, exchange rates can also be tuned by forming micelles at higher pH values and therefore de facto changing the number of charges per dendrimer. Forming generation 6 based micelles at pH 7.8 and thereby decreasing the charge per G6 dendrimer by about 25% results in borderline micelles with very slow exchange kinetics, whereas no dynamics were detected at pH 7.0. It is likely that not only the number of charges but also the charge density, i.e. the charge per volume, influences the exchange rate. Current efforts are under way to corroborate the dynamics with computational models linking the charge density of the dendrimers with that of the block copolymers. By changing the pH after micelle formation from 7.8 to 7.0, thereby increasing the charge per dendrimer, micelle rearrangements are induced in which dendrimers are released from the micelle cores, which has been unambiguously proven by DOSY NMR measurements. This dendrimer release at the same time initiated exchange of generation 6 dendrimers between micelles. In fact, exchange of kinetically trapped generation 6 dendrimers from micelle cores can also be initiated by addition of free dendrimer to a micelle solution. Having a detailed understanding and control of the dendrimer exchange is important for e.g. drug delivery applications, and we believe that this is an important step forward toward the designs of responsive materials. For example, various strategies can be designed that allow for different time scales for release and/or exchange by playing with the various relevant parameters of the toolbox.

## Experimental Section

### Materials

Amine-terminated polyamidoamino dendrimers, generations 4, 5, 6, were purchased from Dendritech Inc., MI, USA, as a 5 wt % methanolic solution and used as a 2.89 mM aqueous solution based on primary amine content. pMAA_64_-*b*-pEO_885_ (*M*_w_/*M*_n_ = 1.15) was obtained from Polymer Sources Inc., Canada, and used as a 5 mM aqueous solution based on carboxylic acid content. Fluorescein isothiocyanate, rhodamine B isothiocyanate, 3-(*N*-morpholino)propanesulfonic acid (MOPS) sodium salt, and 1 M NaOH solutions were obtained from Sigma-Aldrich.

### Characterization

DLS was done on a Malvern Zetasizer Nano S instrument equipped with a laser operating at 633 nm. NMR spectra of the mixture of generation 6 PAMAM-NH_2_ and PAMAM-OH in D_2_O were obtained on a Bruker Avance III spectrometer operating at 500 MHz for 1 H, equipped with a 5 mm TXI probe. NMR spectra of generation 6 PAMAM-NH_2_ in MOPS buffer and D_2_O were obtained on a Bruker Avance III spectrometer operating at 600 MHz for 1 H, equipped with a 2.5 mm SEI probe. All other NMR spectra, of nonfunctionalized free dendrimers and micelles in D_2_O, were obtained on a Bruker NEO spectrometer operating at 28.2 T (1200 MHz 1H-Larmor frequency), equipped with a 3 mm room-temperature probe. All NMR spectra were analyzed using Topspin. Diffusion coefficients were obtained by fitting the attenuated signals of the series of 1D DOSY spectra. Fluorescence emission spectra were acquired on a Cary Eclipse spectrophotometer. Absorbance spectra were obtained on a Shimadzu UV-1900 UV–vis spectrophotometer.

### Dendrimer Functionalization

Functionalization was done as described previously.^32^ Briefly, 2 mg of PAMAM dendrimers of generation 4, 5, or 6 in methanol were added to a vial. Amounts of 4–6 mol equiv of fluorescein isothiocyanate (FITC) or rhodamine B isothiocyanate (RITC) with respect to the dendrimers were dissolved in 1 mL of methanol and added dropwise to the dendrimer solution, with mixing. The mixture was stirred at room temperature for several hours and covered with aluminum foil. Afterward, the solvent was evaporated under reduced pressure and the dried product was dissolved in demineralized water. Unreacted FITC or RITC was removed by dialysis against demineralized water using a dialysis membrane with a molecular cutoff of 3.5–5 kDa, covered with aluminum foil. The purified functionalized dendrimers were again dried under reduced pressure and dissolved in D_2_O for NMR analysis. The dendrimer concentration was determined with NMR using the internal standard TMSP-D4, and the functionalization degree was calculated using UV–vis.

### Dendrimicelle Formation

Dendrimicelles were formed under charge stoichiometric conditions following an established protocol.^45^ For this, 20 μL of an aqueous 2.89 mM PAMAM dendrimer solution (charge concentration based on surface groups) was added to 20 μL of 0.2 M MOPS buffer at pH 7. This solution was dissolved in 149 μL of demineralized water. Afterward, 11 μL of an aqueous 5 mM pMAA_64_-*b*-pEO_885_ solution (charge concentration based on −COO−) was added during sonication for 2 min. Samples were left to equilibrate overnight before characterization and exchange experiments. To make stoichiometric micelles at pH 7.8, only 8.25 μL of the block copolymer solution was added to compensate for fewer positive charges on the dendrimers.

### Determination of Critical Micelle Concentration

The critical micelle concentration was determined with dynamic light scattering by measuring micelles at different concentrations. The scattering intensity was converted to the excess Rayleigh ratio according to the equation
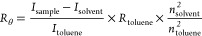
4with *R*_θ_ being the excess Rayleigh ratio at scattering angle θ, *R*_toluene_ the known Rayleigh scattering ratio of toluene (1.35 × 10^–3^ m^–1^), *n*_solvent_ and *n*_toluene_ the refractive indices of water and toluene (1.333 and 1.497, respectively), and *I*_sample_, *I*_solvent_, and *I*_toluene_ the scattering intensities of the sample, water and toluene, respectively.

By plotting the excess Rayleigh ratio versus the total polymer concentration (block copolymer + dendrimer) and a linear fitting, the CMC is determined from the intersection with the *x* axis.

### Exchange Experiment

For exchange experiments, dendrimicelles containing only the donor (dendrimers functionalized with FITC) or only the acceptor (dendrimers functionalized with RITC) were prepared according to the aforementioned protocol. For all samples the charge concentration of the dendrimers was kept the same. The next day, the samples were diluted five times to avoid an inner filter effect. To measure the exchange, half of the only-donor and half of the only-acceptor samples were mixed at a 1:1 ratio (500 μL each) and measured immediately after mixing every 1 min for 2 h. The excitation wavelength was 480 nm, and the emission range was 490–700 nm. To calculate the A/D ratio, integrals from 500 to 540 nm (donor, D) and from 575 to 615 nm (acceptor, A) were taken.

As reference sample 1 for the starting point, the other half of each sample (500 μL) was mixed with 500 μL of a dendrimicelle solution containing nonfunctionalized dendrimers. This was done to keep the total volume and the micelle concentration the same. The fluorescence emission spectra of both samples, only-donor and only-acceptor mixed with nonfunctionalized dendrimicelles, were measured separately with an excitation wavelength of 480 nm. The sum of these two spectra was used to calculate the starting A/D ratio for the exchange experiment.

Reference sample 2, containing both the donor and the acceptor within one micelle at a 1:1 ratio, was prepared to determine the maximum A/D ratio showing complete exchange. The fluorescence emission spectrum of this sample was recorded with an excitation wavelength of 480 nm.

To get the α_A/D_ value, the A/D ratios for the exchange sample were then normalized to the A/D ratios from the two reference samples, using eq 3.

For the mixed-generation micelles, both generations are always present in all micelles. The ratio between the two generations is always 1:1, based on the number of dendrimers.

## Supporting Information Available

DLS, UV–vis, fluorescence, and NMR characterization of dendrimers and resulting dendrimicelles, additional exchange experiments for a prolonged time or at different concentrations as well as bleaching control experiments for dendrimer generation 5, and exchange experiments of mixed generations (PDF)
