# Supplementary material for: Controlling Trapping, Release, and Exchange Dynamics of Micellar Core Components
Source: ACS Nano. 2022 Sep 15;16(9):14611–21. doi: 10.1021/acsnano.2c05144 (PMC9527800; doi:10.1021/acsnano.2c05144)
Supplement: Supplementary file 1 — nn2c05144_si_001.pdf [file nn2c05144_si_001.pdf]

Supporting Information for

Controlling Trapping, Release and Exchange Dynamics of Micellar Core Components

Rebecca Kaup[†], Aldrik H. Velders [*,†,‡,§]

†Laboratory of BioNanoTechnology, Wageningen University. Bornse Weilanden 9, 6708 WG Wageningen, The Netherlands.

‡ Interventional Molecular Imaging Laboratory, Department of Radiology, Leiden University Medical Center, 2300 RC Leiden, The Netherlands.

§ Instituto Regional de Investigacion Cientifica Aplicada (IRICA), Universidad de Castilla-La Mancha. Ciudad Real, 13071, Spain.

*E-mail: aldrik.velders@wur.nl

Micelle formation and characterization

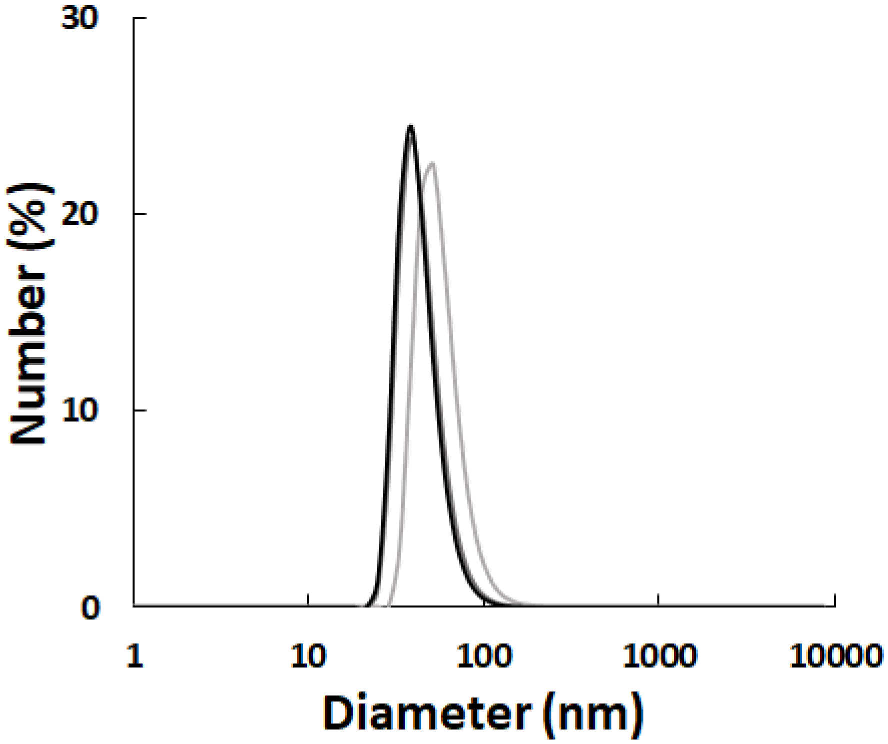

Figure S1: DLS plots of dendrimicelles based on dendrimer generation 4 (light grey), 5 (dark grey) and 6 (black).

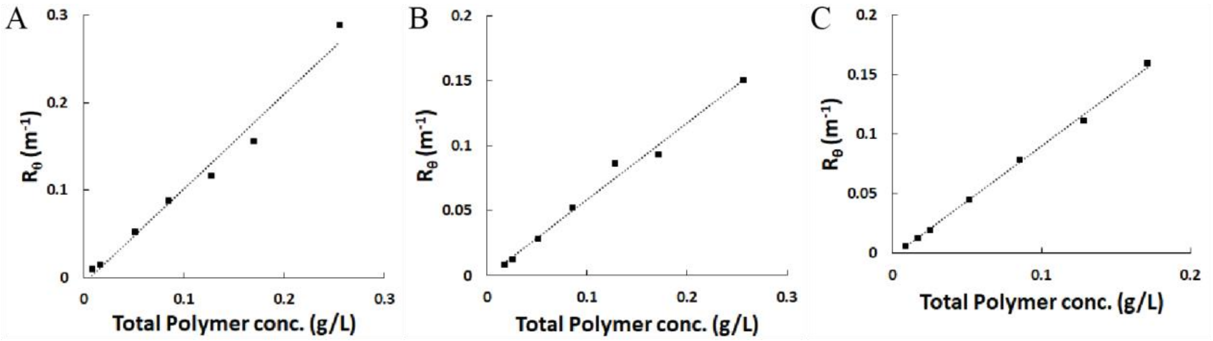

Figure S2: CMC determination of generation 4 (A), generation 5 (B) and generation 6 (C) based micelles. The excess Rayleigh ratio is plotted versus the total polymer concentration. The data points were fitted linearly and the CMC was determined by extrapolation to zero scattering intensity. The CMC values found for G4, G5 and G6 are 6 mg/L, 1 mg/L and < 3 mg/L total polymer concentration, respectively.

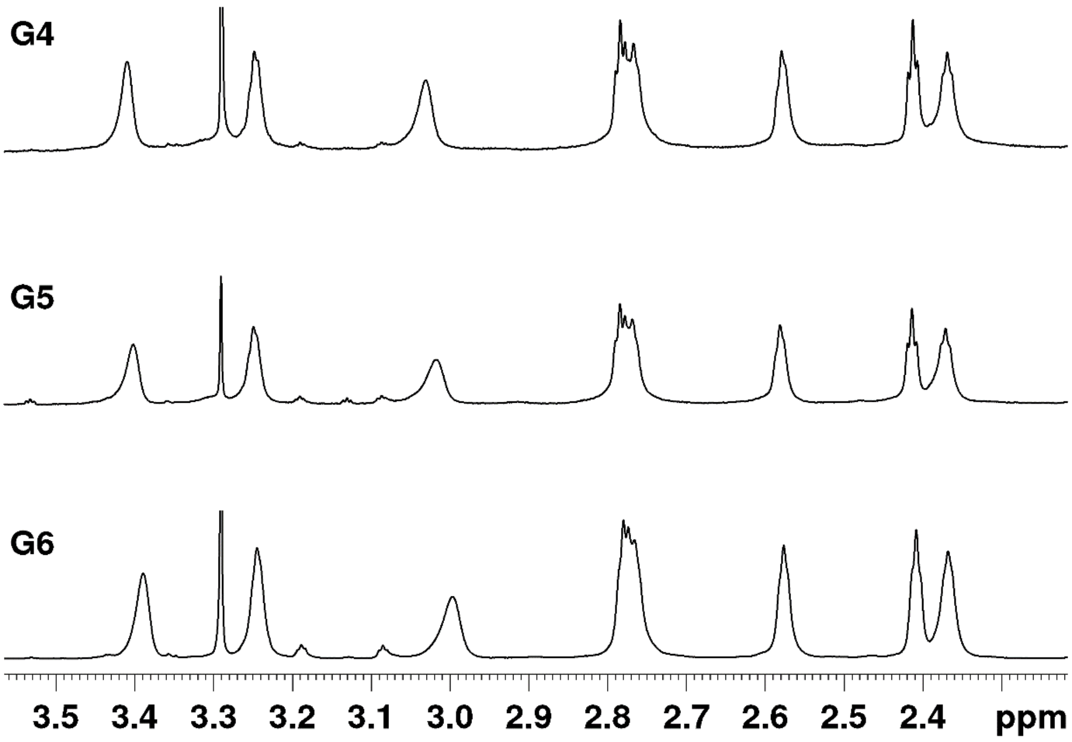

Figure S3: Proton NMR of PAMAM dendrimers generation 4, 5 and 6 in D2O.

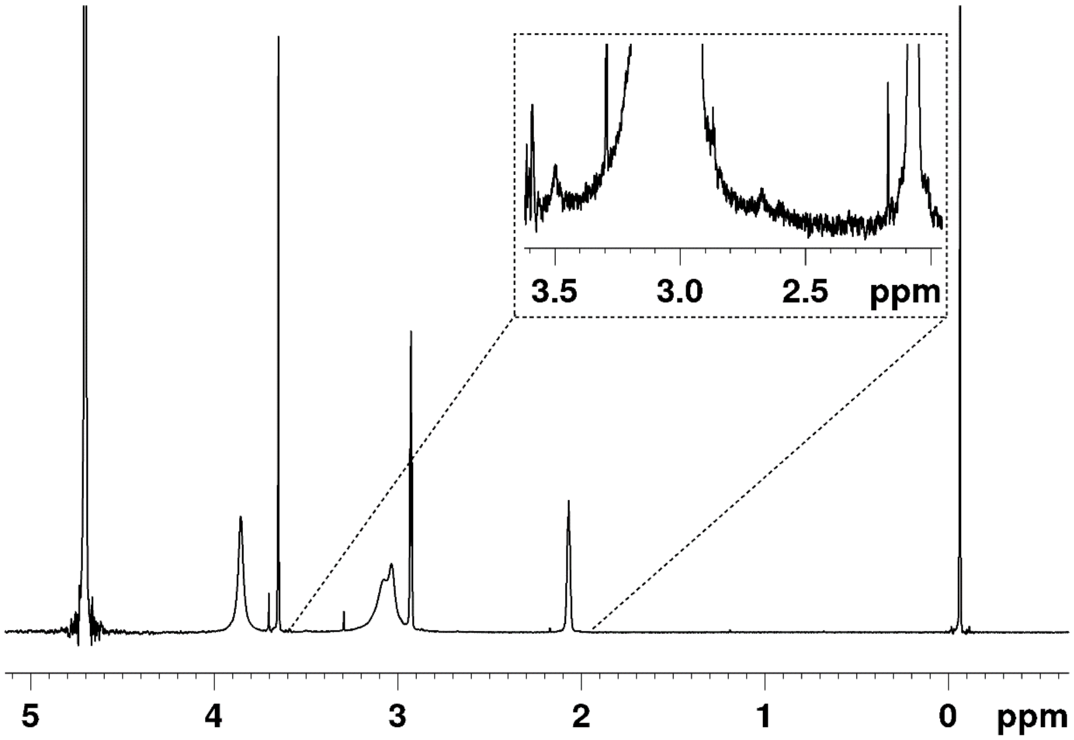

Figure S4: Proton NMR spectrum of generation 4 based micelles. The peaks at 0, 2, 2.9, 3.1, 3.8 and 4.7 are from residual protonated TMSP-D4 (internal standard), MOPS buffer and water, respectively. The peak at 3.6 is from the PEG part of the block copolymer. The insets show the enlarged dendrimer peaks at 2.7 and 3.5 ppm.

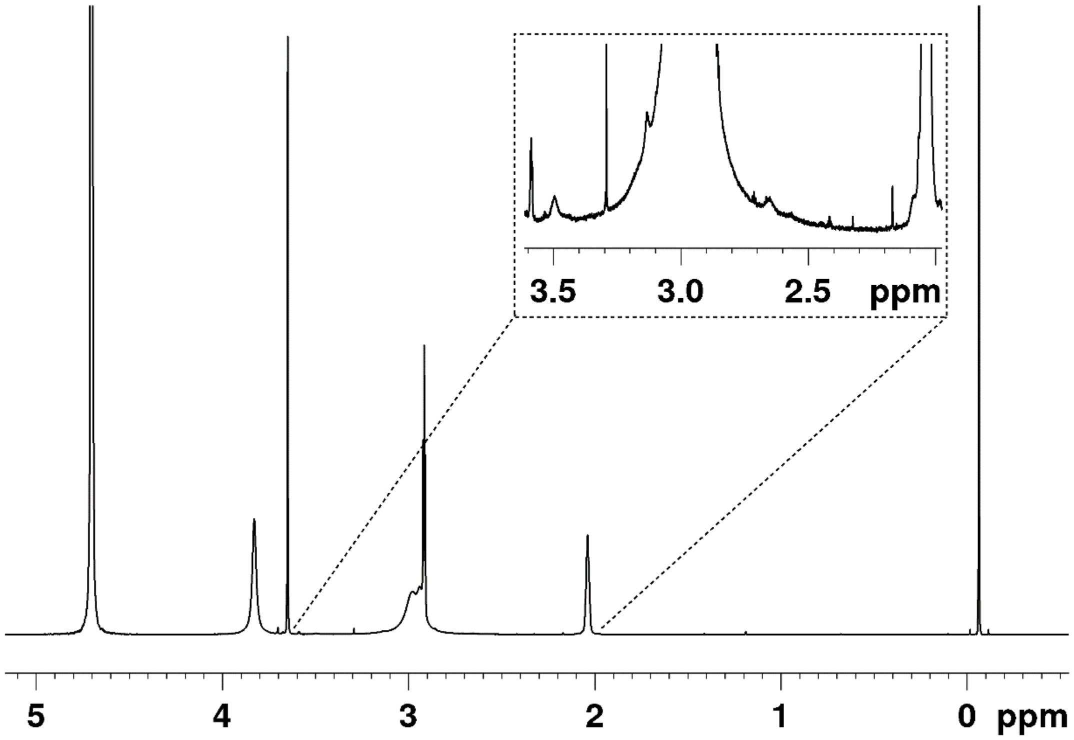

Figure S5: Proton NMR spectrum of generation 5 based micelles. The peaks at 0, 2, 2.9, 3, 3.8 and 4.7 are from residual protonated TMSP-D4 (internal standard), MOPS buffer and water, respectively. The peak at 3.6 is from the PEG part of the block copolymer. The insets show the enlarged dendrimer peaks at 2.7 and 3.5 ppm.

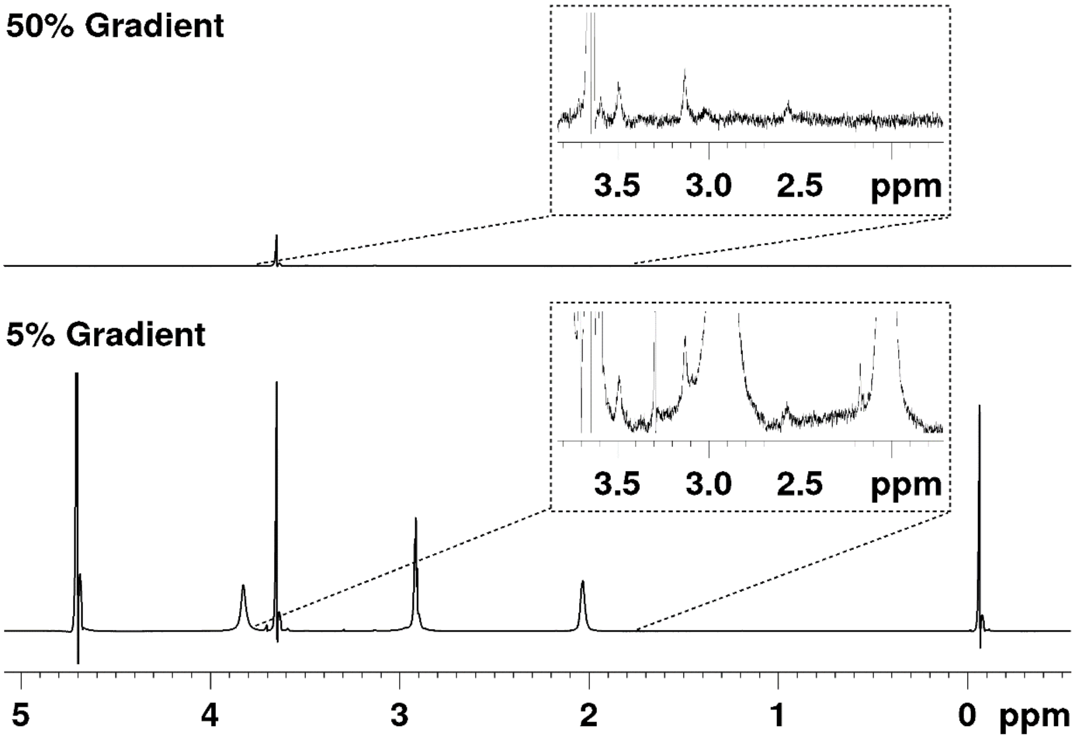

Figure S6: NMR spectrum of generation 5 based micelles with 5% (bottom part) or 50% (upper part) gradient strength. The peaks at 0, 2, 2.9, 3.8 and 4.7 are from residual protonated TMSP-D4 (internal standard), MOPS buffer and water, respectively. The peak at 3.6 is from the PEG part of the block copolymer. The insets show the enlarged dendrimer peaks at 2.7, 3.1 and 3.5 ppm.

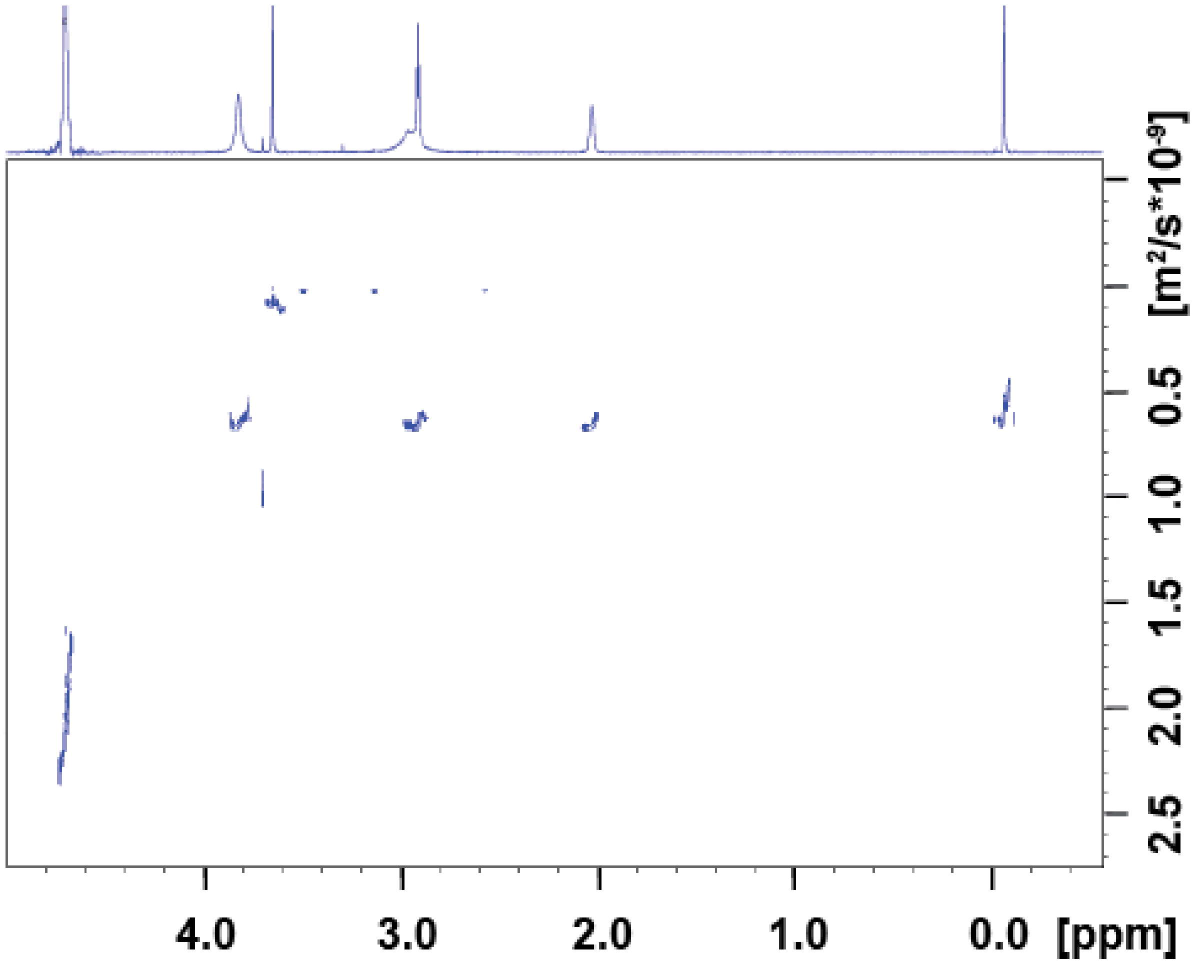

Figure S7: DOSY NMR spectrum of generation 6 based micelles. The peaks at 0, 2, 3, 3.8 are from residual protonated TMSP-D4 (internal standard) and MOPS buffer, respectively. The peaks at 3.6, 3.7 and 4.7 are from the PEG part of the block copolymer, methanol and D2O. The peaks at 2.6, 3.1 and 3.5 ppm are from the dendrimers.

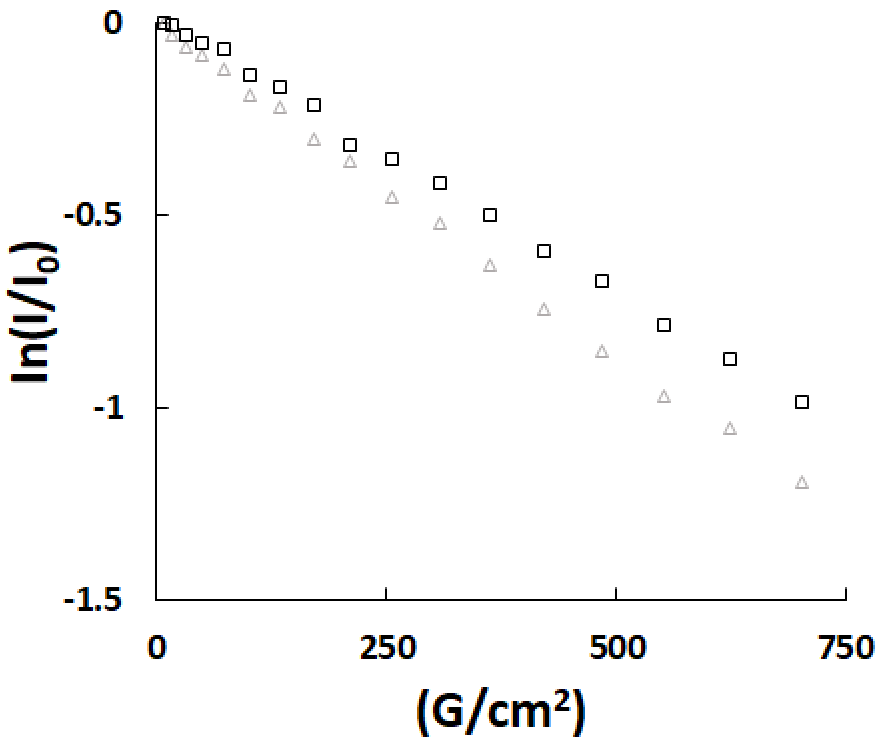

Figure S8: T1/T2 relaxation from a DOSY NMR experiment of generation 6 PAMAM-NH_2 dendrimers (black squares) mixed with generation 6 PAMAM-OH dendrimers (grey triangles). The diffusion coefficients of PAMAM-NH2 and PAMAM-OH are $4.1*10^{-11}$ m^2/s and $4.86*10^{-11}$ m^2/s, respectively. Peaks at 2.8 ppm (-NH_2) and 3.5 ppm (-OH) were taken for analysis.

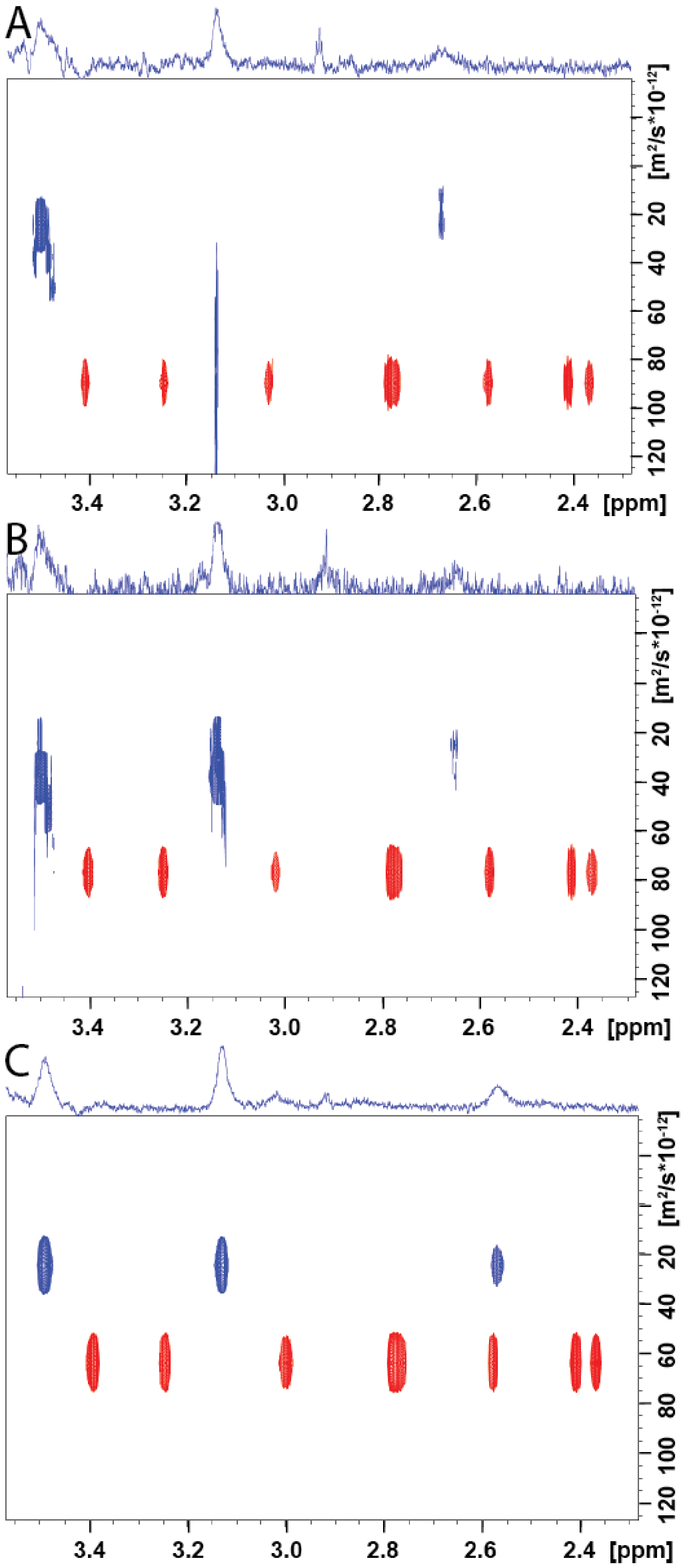

Figure S9: DOSY spectra of dendrimers encapsulated in micelles (blue) or free in solution (red) for (A) generation 4, (B) generation 5 and (C) generation 6. The 1D spectra were taken with a gradient strength of 30 %.

Characterization of functionalized dendrimers and resulting micelles

Table S1: Overview of PAMAM dendrimers generation 4, 5 and 6 functionalized with fluorescein (FITC) or rhodamine B (RITC). Based on UV-Vis measurements, the number of fluorophores per dendrimer and the quantum yield (QY) of the functionalized dendrimers were determined.

Dendrimer Generation	Average FITC per dendrimer	QY (FITC)	Average RITC per dendrimer	QY (RITC)
4	3	0.07	2	0.03
5	4	0.15	2	0.06
6	4	0.21	2	0.06

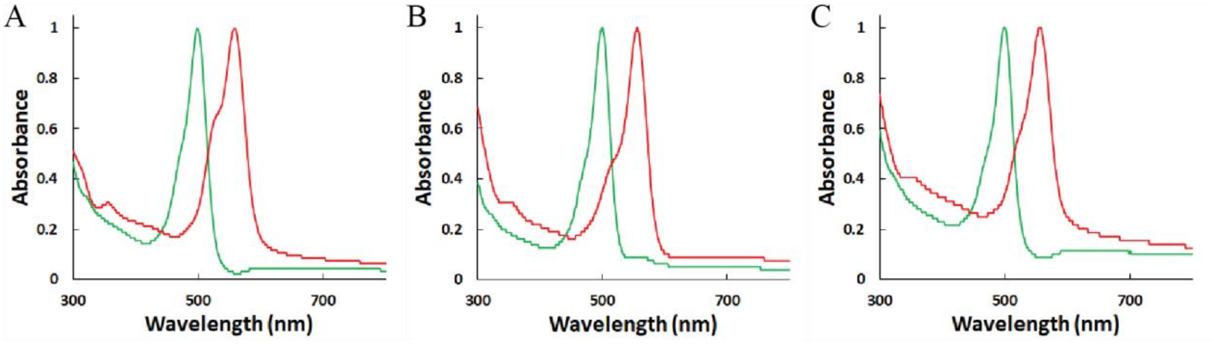

Figure S10: UV-Vis spectra of dendrimers generation 4 (A), 5 (B) and 6 (C), functionalized with FITC (green) or RITC (red).

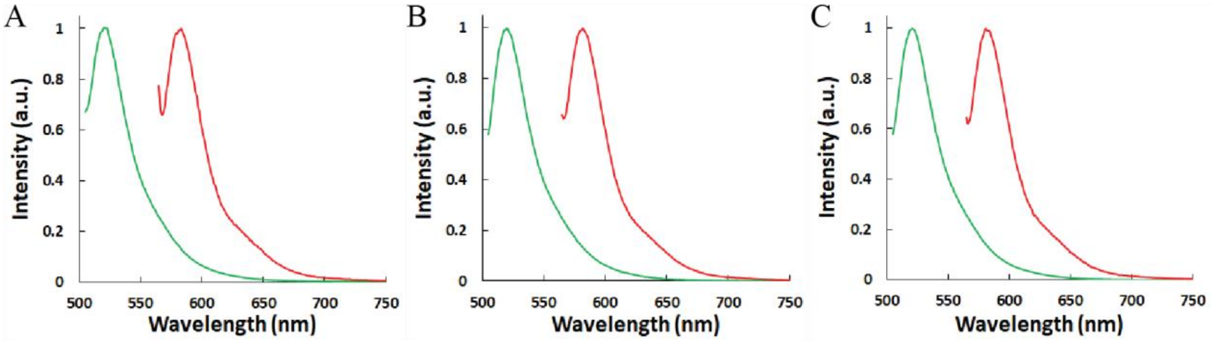

Figure S11: Emission spectra of dendrimers generation 4 (A), 5 (B) and 6 (C), functionalized with FITC (green) or RITC (red). Excitation wavelengths were λ = 499 nm for fluorescein functionalized dendrimers and λ = 558 nm for rhodamine B functionalized dendrimers.

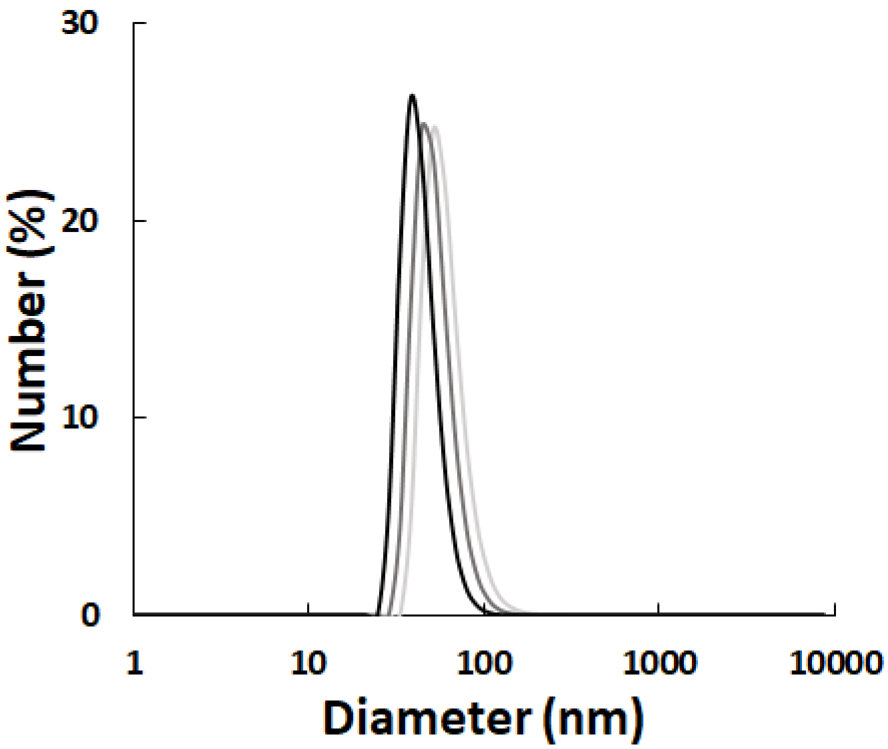

Figure S12: DLS plots of dendrimicelles based on dendrimer generation 4 (light grey), 5 (dark grey) and 6 (black). The dendrimicelles contain dendrimers labelled with either fluorescein or rhodamine at a 1:1 ratio.

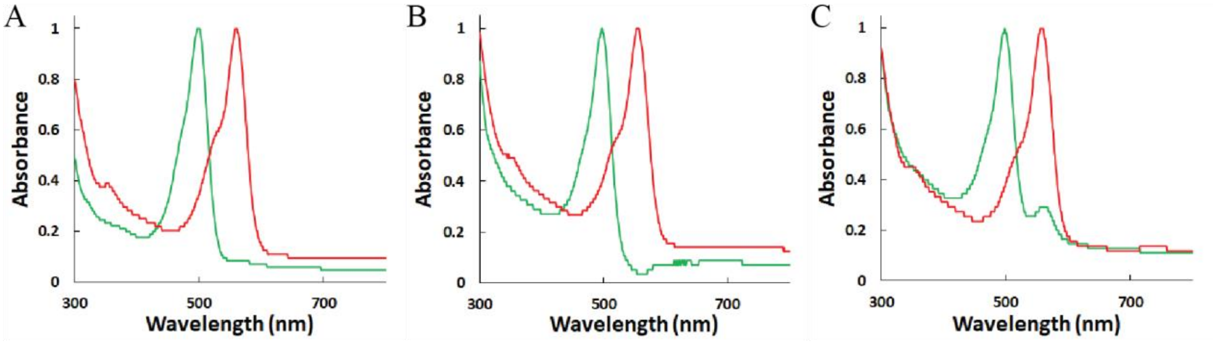

Figure S13: UV-Vis spectra of micelles made from fluorophore functionalized dendrimers generation 4 (A), 5 (B) and 6 (C). Micelles containing dendrimers functionalized with FITC or RITC are depicted in green and red, respectively.

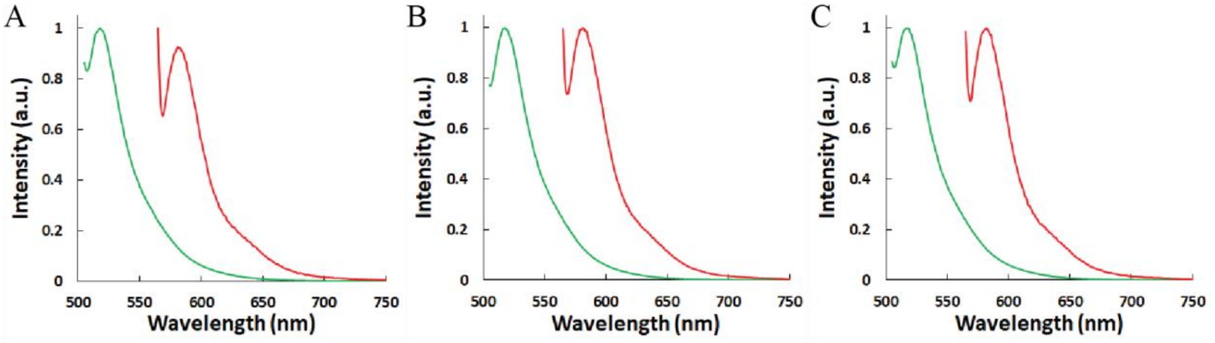

Figure S14: Emission spectra of micelles made from fluorophore functionalized dendrimers generation 4 (A), 5 (B) and 6 (C). Micelles containing dendrimers functionalized with FITC or RITC are depicted in green and red, respectively. Excitation wavelengths were λ = 499 nm for fluorescein functionalized dendrimers and λ = 558 nm for rhodamine B functionalized dendrimers.

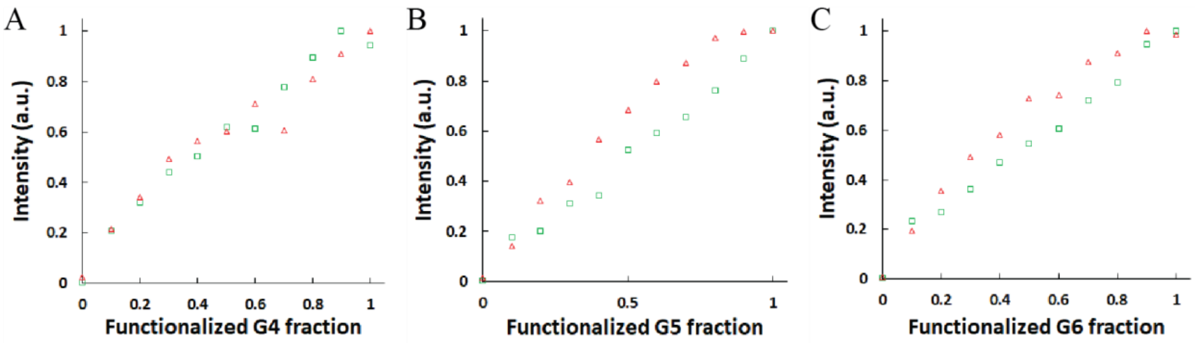

Figure S15: Fluorescence intensity of micelles containing (A) generation 4, (B) generation 5 or (C) generation 6 dendrimers. Micelles containing dendrimers functionalized with FITC and non-functionalized dendrimers at different ratios are depicted in green. Micelles containing dendrimers functionalized with RITC and non-functionalized dendrimers at different ratios are shown in red.

Exchange of dendrimers between micelles

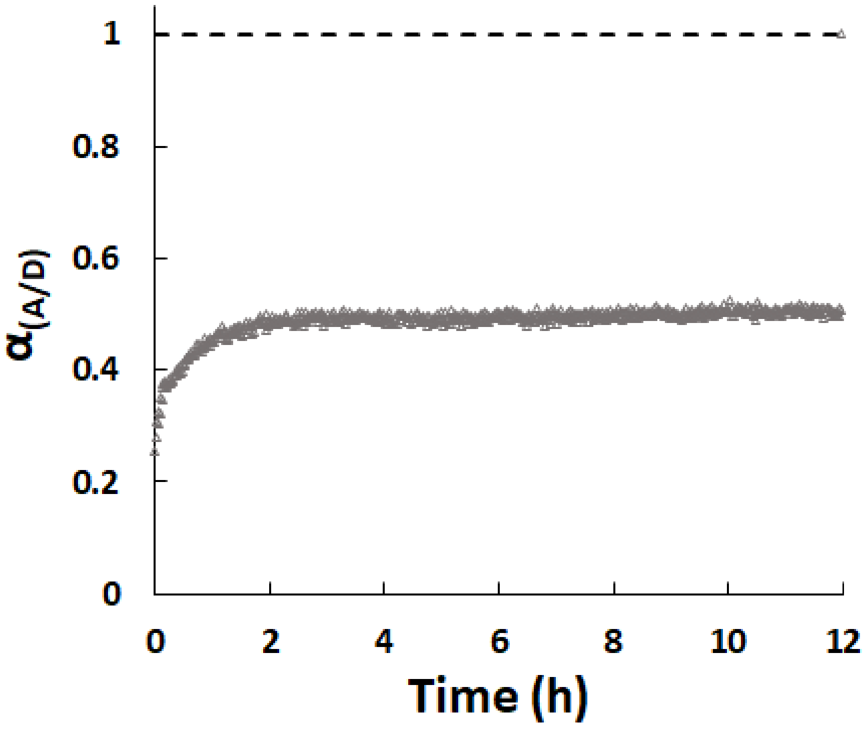

Figure S16: $\alpha_{(A/D)}$ over time for Generation 5 based micelles. For this only donor (FITC) and only acceptor (RITC) micelles were mixed at a 1:1 ratio. The first point at $\alpha_{(A/D)}$ = 0 corresponds to the combined donor only and acceptor only spectrum. The dashed line at $\alpha_{(A/D)}$ = 1 corresponds to the spectrum of micelles containing both donor and acceptor, representing complete exchange. Excitation wavelength was 480 nm.

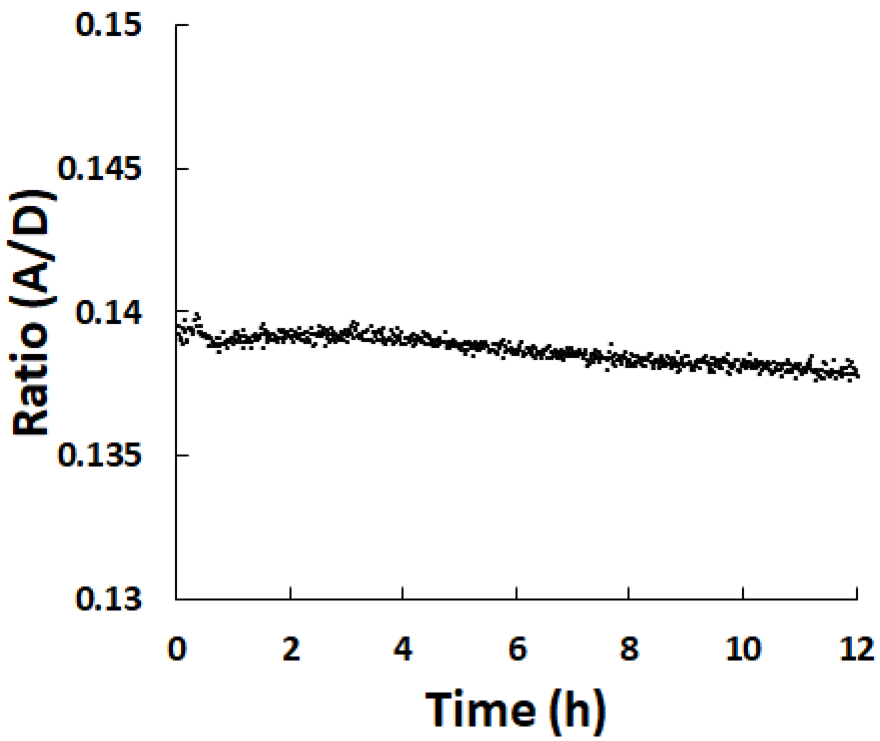

Figure S17: Ratio (A/D) over time for Generation 5 based micelles. For this dendrimers labelled with donor (FITC) and dendrimers labelled with acceptor (RITC) were mixed at a 1:1 ratio before micelle formation. The Ratio (A/D) stays stable for four hours. After four hours the ratio decreases, probably due to bleaching effects. Excitation wavelength was 480 nm.

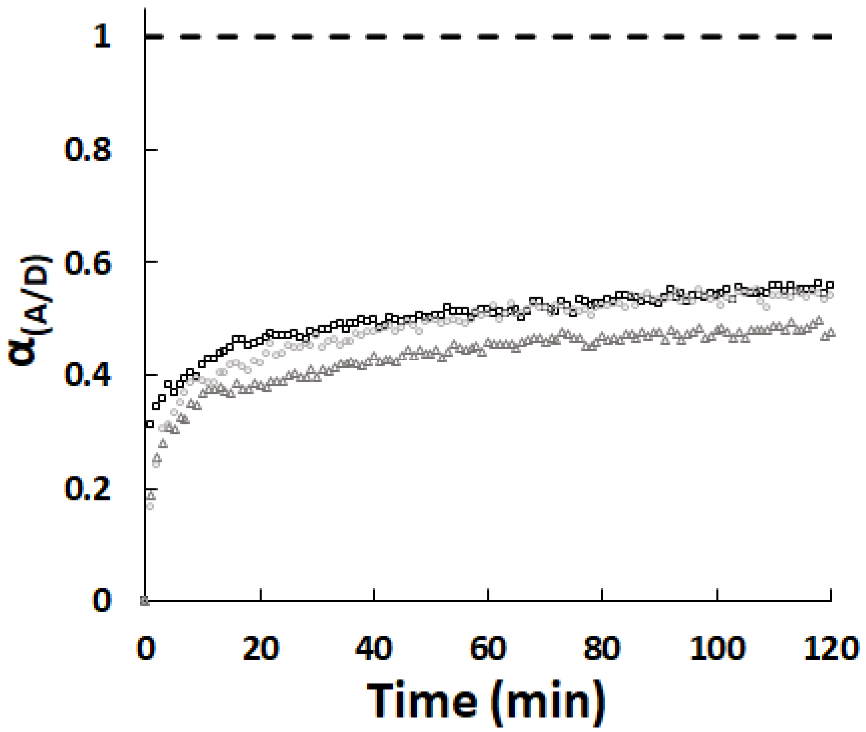

Figure S18: $\alpha_{(A/D)}$ over time for Generation 5 based micelles at different concentrations, i.e. dendrimer concentration is 0.9 μM (black squares), 0.45 μM (dark grey triangles) and 0.23 μM (light grey circles). For this only donor (FITC) and only acceptor (RITC) micelles were mixed at a 1:1 ratio. The first point at $\alpha_{(A/D)}$ = 0 corresponds to the combined donor only and acceptor only spectrum. The dashed line at $\alpha_{(A/D)}$ = 1 corresponds to the spectrum of micelles containing both donor and acceptor, representing complete exchange. Excitation wavelength was 480 nm.

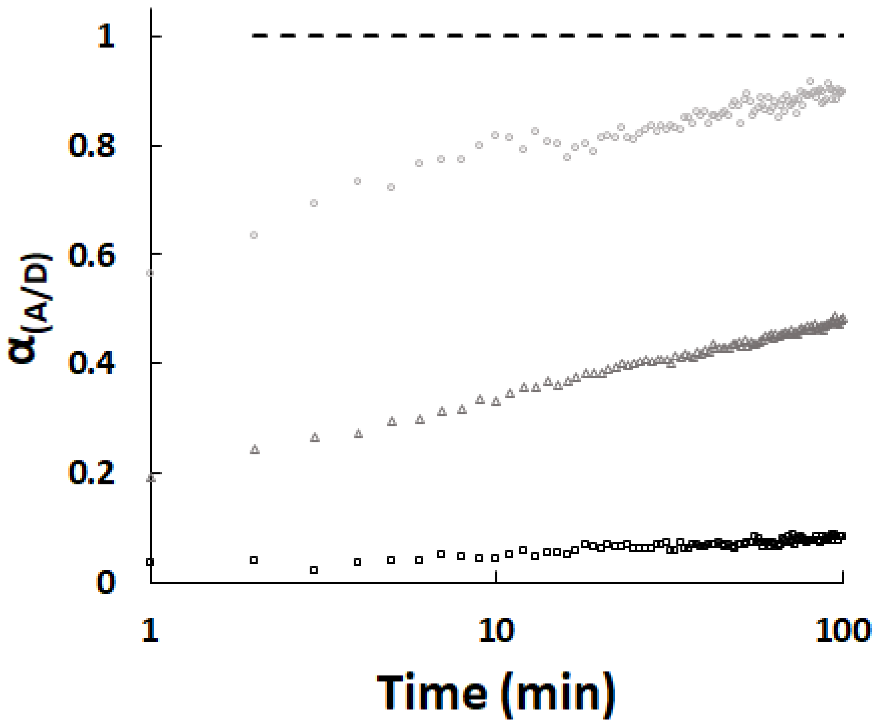

Figure S19: $\alpha_{(A/D)}$ over time for Generation 4, 5 and 6 based micelles. For this only donor (FITC) and only acceptor (RITC) micelles were mixed at a 1:1 ratio. The first point at $\alpha_{(A/D)}$ = 0 corresponds to the combined donor only and acceptor only spectrum. The dashed line at $\alpha_{(A/D)}$ = 1 corresponds to the spectrum of micelles containing both donor and acceptor, representing complete exchange. Excitation wavelength was 480 nm. (These data are from figure 2 from the main text, but plotted on a logarithmic scale.)

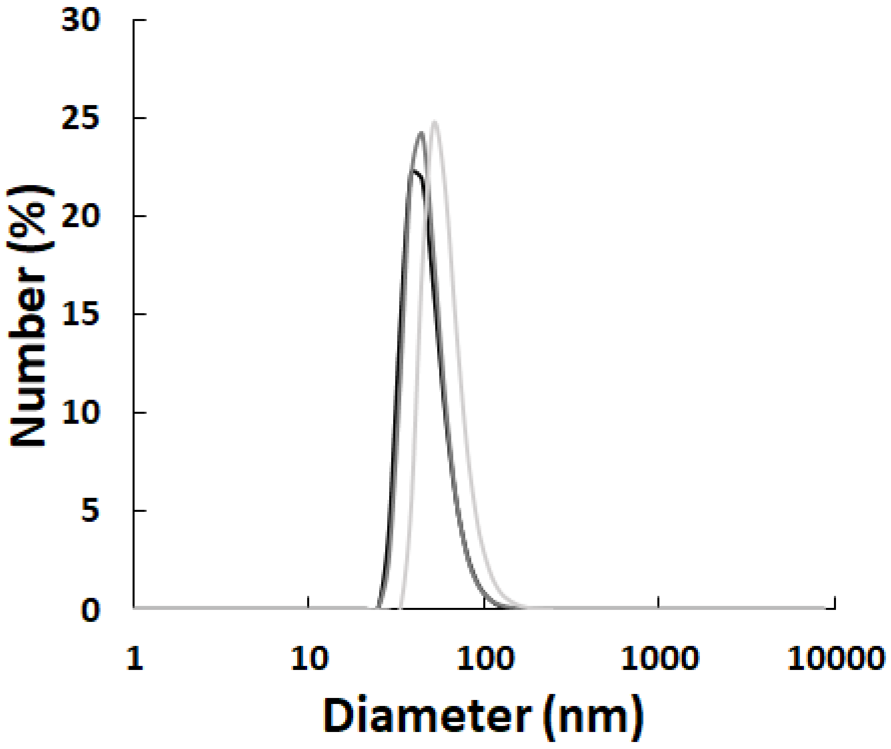

Figure S20: DLS plots of dendrimicelle based on two different dendrimer generations within the same micellar core. Generation 5 + 4 (light grey), 6 + 4 (dark grey) and 6 + 5 (black) based micelles.

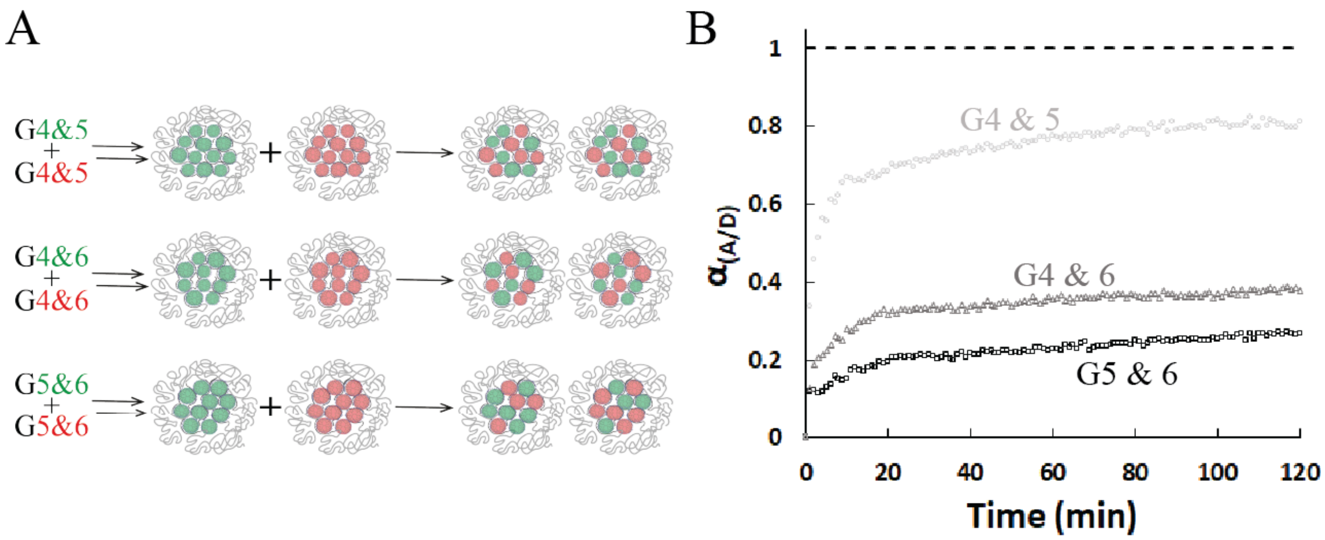

Figure S21: A. Cartoon showing three different dendrimicelle samples. Generations 4 + 5, generations 4 + 6 or generations 5 + 6 dendrimers are encapsulated in either only donor (green) or only acceptor (red) micelles. Both generations are present in the only donor and in the only acceptor micelle. The different dendrimer generations are indicated by different sizes of the dendrimers. The number of dendrimers increases with decreasing dendrimer generation. Mixing of micelles at a 1:1 ratio, leads to exchange of dendrimers between micelles resulting in FRET as both donor and acceptor end up in the same micelle core. B. Percentage of max FRET over time for Generation 4 + 5 (light grey, circles), generation 4 + 6 (dark grey, triangles) and generation 5 + 6 (black, squar es). The first point of each line at $\alpha_{(A/D)} = 0$ corresponds to a combined spectrum of donor only and acceptor only micelles. The dashed line at $\alpha_{(A/D)} = 1$ corresponds to the spectrum of premixed micelles containing both donor and acceptor, representing complete exchange. Excitation wavelength was 480 nm. (For the maximal FRET it was assumed that also generation 6 can exchange between micelles).

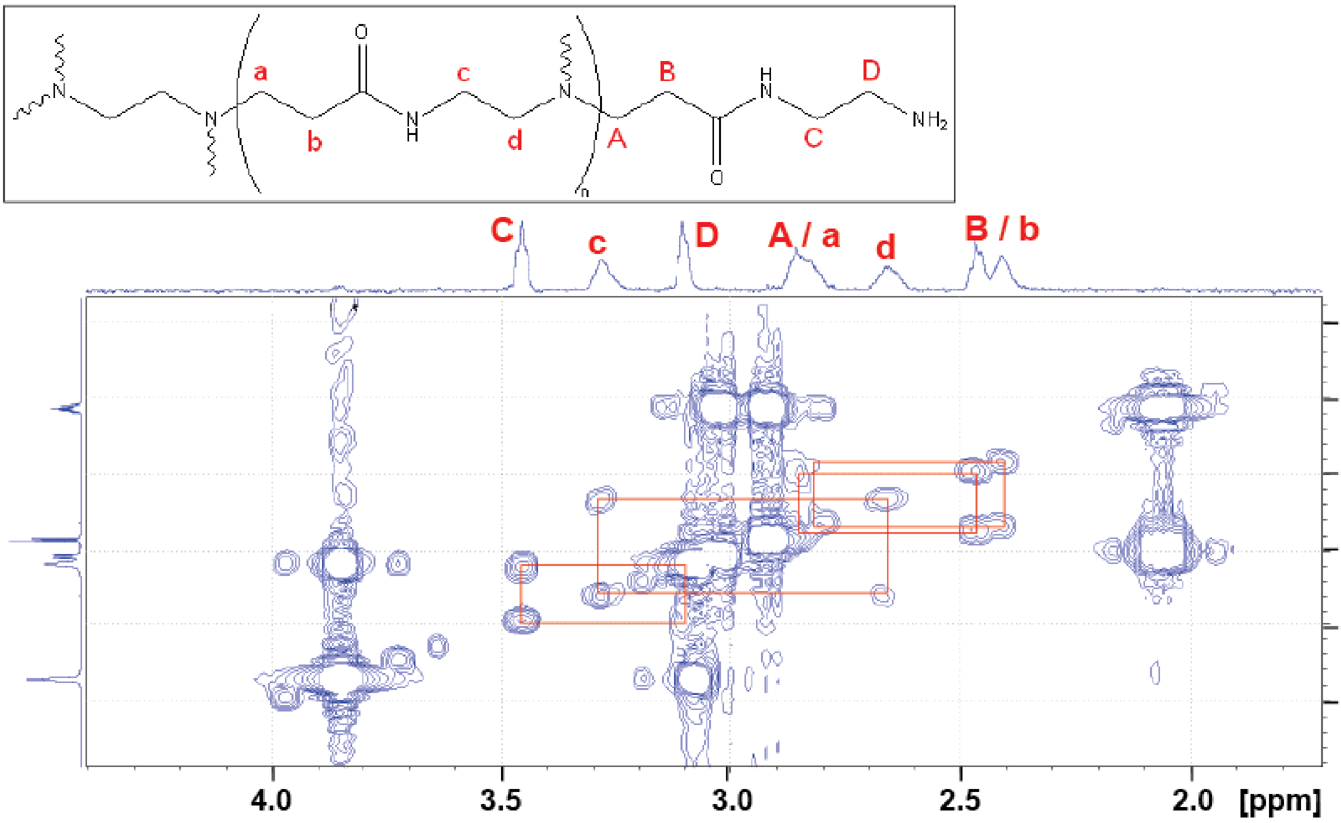

Figure S22: Top part: Chemical structure of a PAMAM dendrimer. Bottom part: COSY plot of PAMAM dendrimers in 0.2 M MOPS buffer in D_2O at pH 7.0. The 1 D spectrum on the left shows the proton spectrum with mainly the buffer peaks. The 1 D spectrum on top shows the DOSY-filtered spectrum with only the dendrimer peaks. The dendrimer crosspeaks are connected with red boxes. Using this spectrum the dendrimer peaks were assigned following previous NMR assignments done by us,[1] and others.[2]

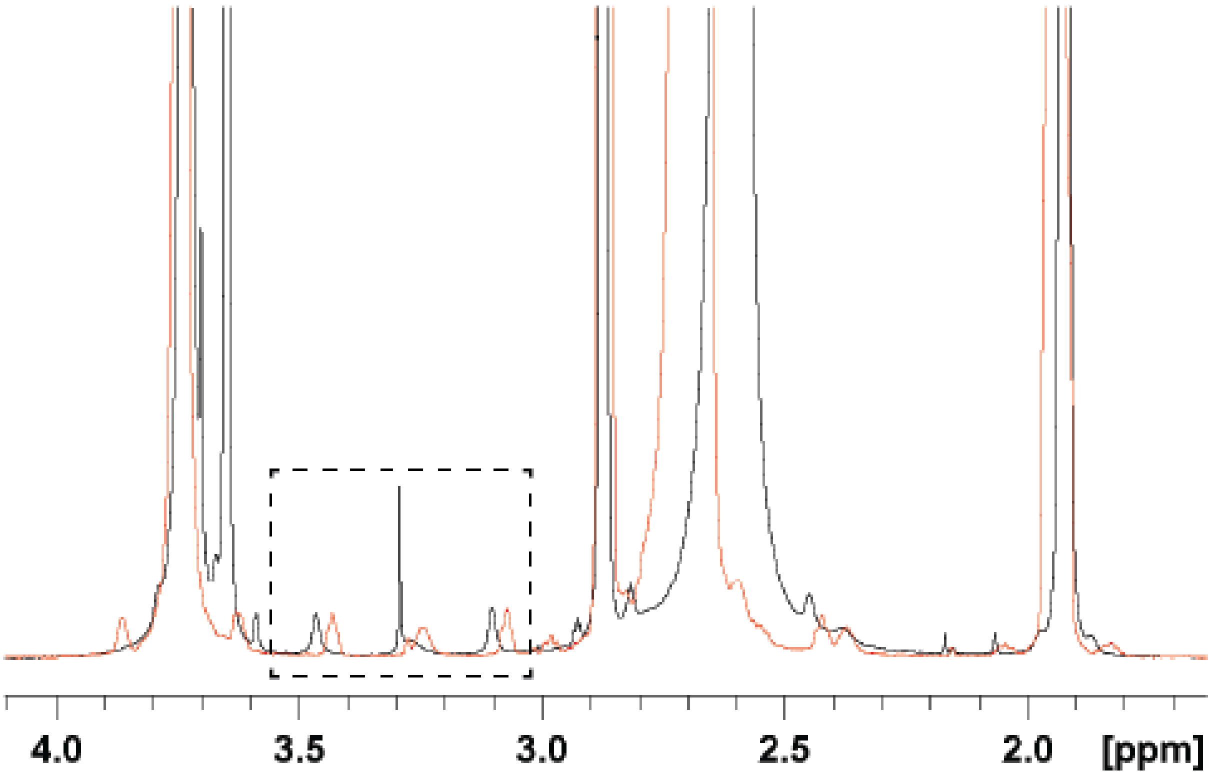

Figure S23: Proton NMR spectra in D2O of G6-based dendrimicelles formed stoichiometric at pH 7.8 (black) and of G6-NH2 PAMAM dendrimers at pH 7.8 free in solution (red). For discussions, the focus Is on the dendrimer peaks highlighted by the black box.

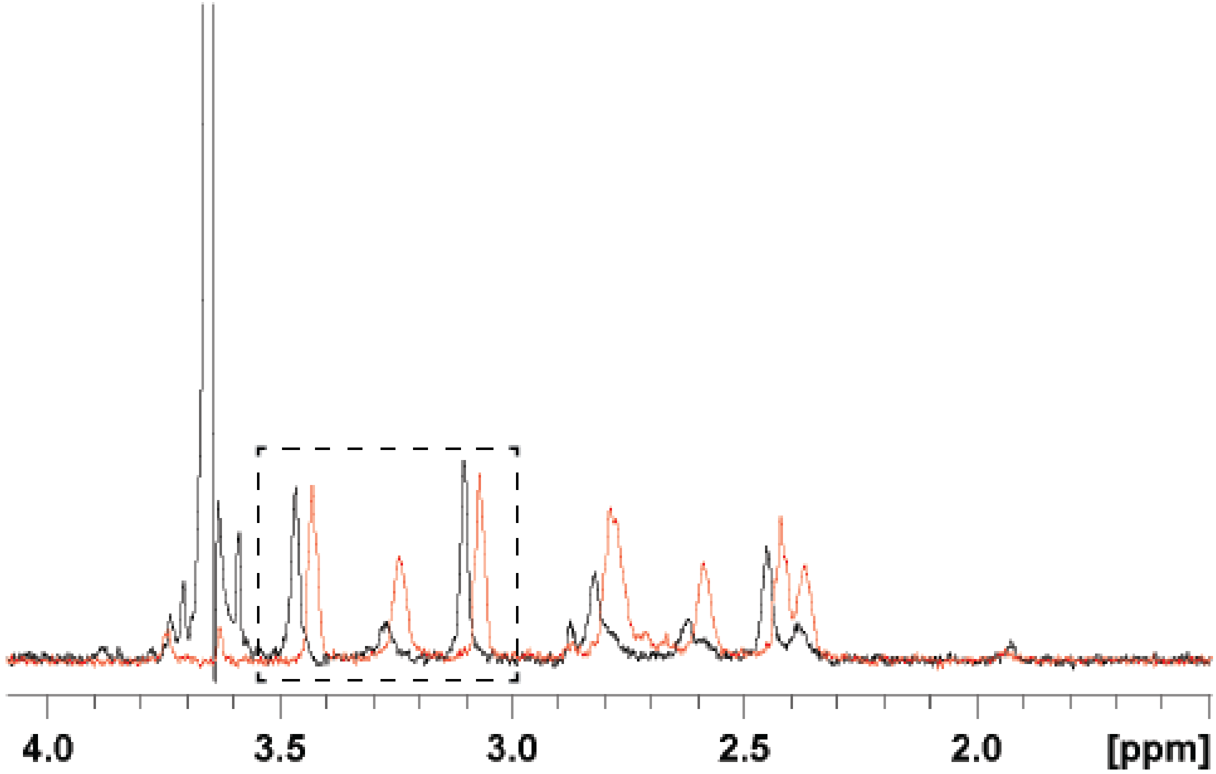

Figure S24: DOSY filtered NMR spectra with 30% gradient strength in D2O of G6-based dendrimicelles formed stoichiometric at pH 7.8 (black) and of G6-NH2 PAMAM dendrimers at pH 7.8 free in solution (red). For discussions, the focus Is on the dendrimer peaks highlighted by the black box.

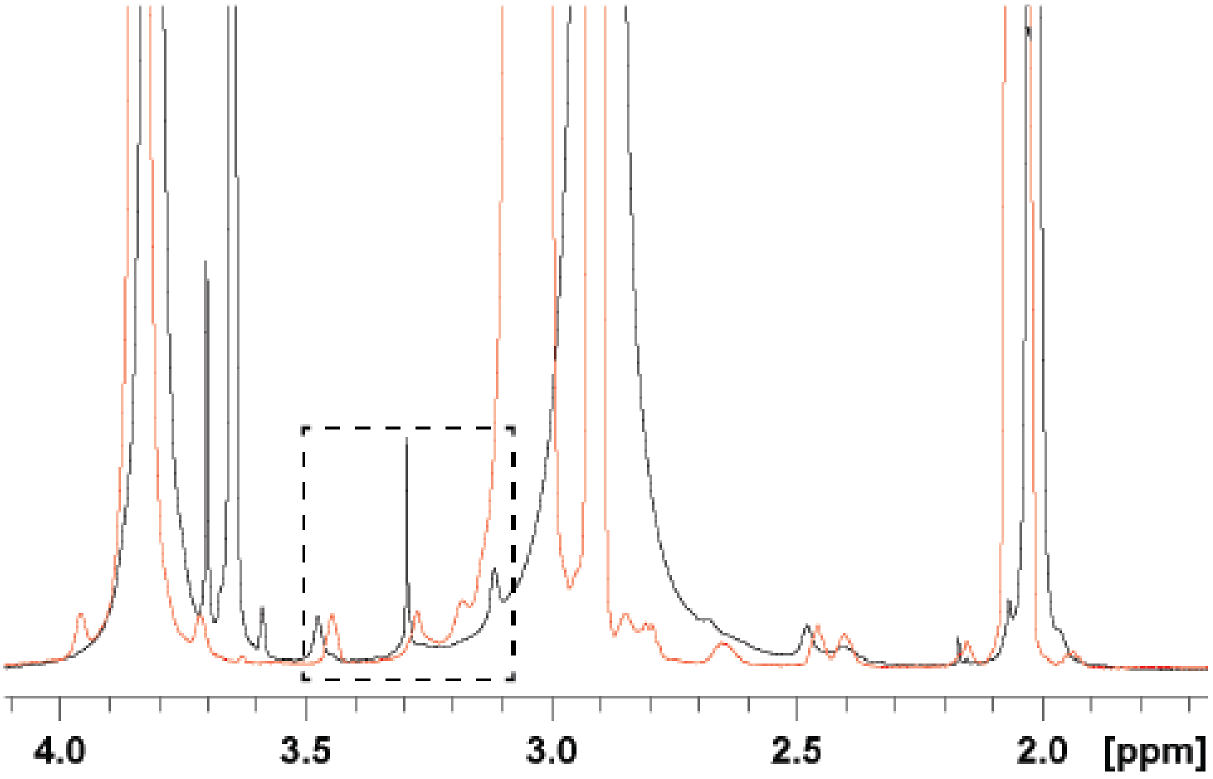

Figure S25: Proton NMR spectra in D2O of G6-based dendrimicelles formed stoichiometric at pH 7.8 after changing the pH to 7.0 (black) and of G6-NH2 PAMAM dendrimers at pH 7.0 free in solution (red). For discussions, the focus Is on the dendrimer peaks highlighted by the black box.

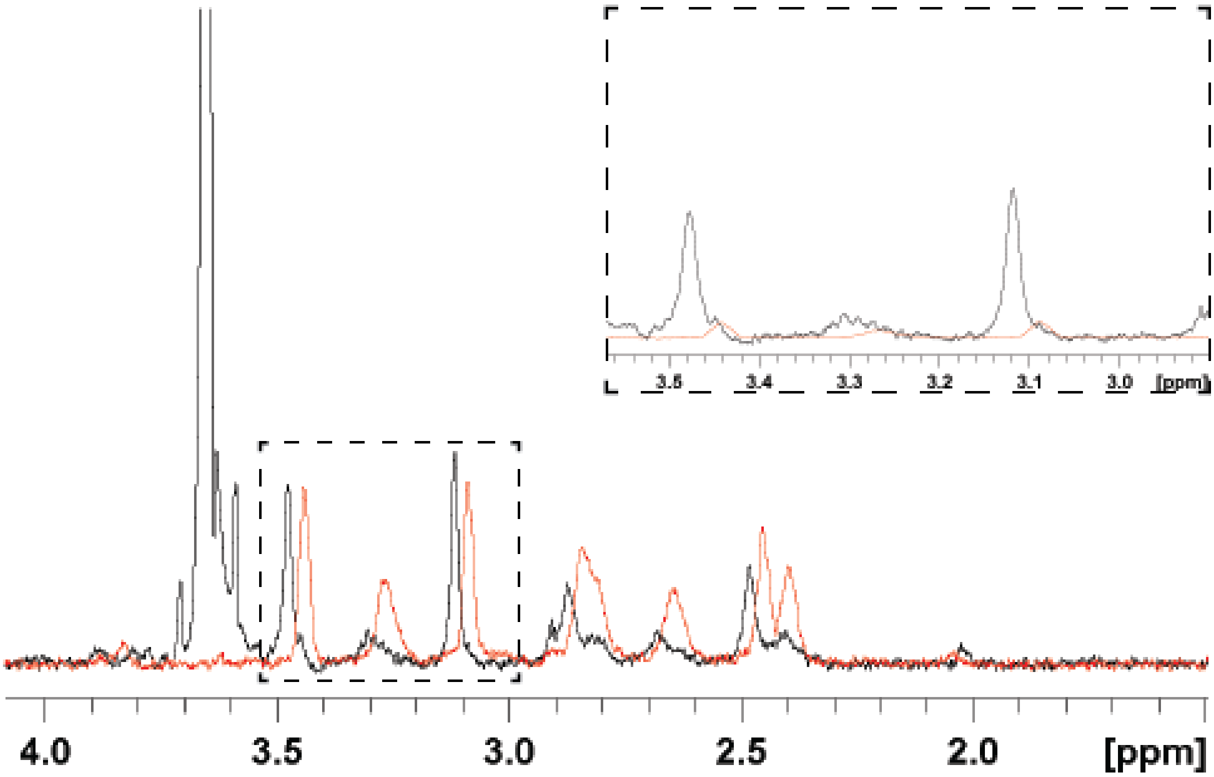

Figure S26: DOSY filtered NMR spectra with 30% gradient strength in D2O of G6-based dendrimicelles formed stoichiometric at pH 7.8 after changing the pH to 7.0 (black) and of G6-NH2 PAMAM dendrimers at pH 7.0 free in solution (red). For discussions, the focus Is on the dendrimer peaks highlighted by the dashed black box. The peaks in the box are enlarged in the upper corner with the peak intensity of the free dendrimers adjusted to the peak intensity of released dendrimers in the micelle sample (3.44 ppm)

Table S2: DLS data of G6-based dendrimicelles formed stoichiometrically at pH 7.8 before and after changing the pH to 7.0.

Sample	Diameter (nm)	Scattering Intensity (kcps)
G6-based dendrimicelle at pH 7.8 (stoichiometric)	56	8172
G6-based dendrimicelles after changing pH from 7.8 to 7.0	53	7394

References:

[1] Gomez, M. V.; Guerra, J.; Velders, A. H.; Crooks, R. M., NMR Characterization of Fourth-Generation PAMAM Dendrimers in the Presence and Absence of Palladium Dendrimer-Encapsulated Nanoparticles. *J. Am. Chem. Soc.* **2009,** *131* (1), 341-350.

[2] Bohme, U.; Klenge, A.; Hanel, B.; Scheler, U., Counterion Condensation and Effective Charge of PAMAM Dendrimers. *Polymers* **2011,** *3* (2), 812-819.
